# Selection and Phenotypic Plasticity Shape Plant Performance in a Grassland Biodiversity Experiment

**DOI:** 10.1002/ece3.71117

**Published:** 2025-03-13

**Authors:** Francesca De Giorgi, Walter Durka, Yuanyuan Huang, Bernhard Schmid, Christiane Roscher

**Affiliations:** ^1^ Department of Physiological Diversity Helmholtz Centre for Environmental Research – UFZ Leipzig Germany; ^2^ German Centre for Integrative Biodiversity Research (iDiv) Halle‐Jena‐Leipzig Leipzig Germany; ^3^ Department of Community Ecology Helmholtz Centre for Environmental Research – UFZ Halle Germany; ^4^ Institute of Biology, Experimental Interaction Ecology Leipzig University Leipzig Germany; ^5^ Remote Sensing Laboratories, Department of Geography University of Zürich Zürich Switzerland

**Keywords:** adaptation, biodiversity experiment, eco‐evolutionary dynamics, phenotypic responses, phytometer experiment, plant–soil feedback

## Abstract

The increasing strength of positive biodiversity effects on plant community productivity, observed in long‐term biodiversity experiments, relates to mixed responses at the species level. However, it is still not well understood if the observed mixed responses are adaptations to the different selection pressures in plant communities of different diversity or plastic adjustments. We conducted a transplant experiment for nine plant species in a 17‐year‐old biodiversity experiment (Jena Experiment). We used offspring of plants *selected* in the biodiversity experiment and from plants without selection in the experiment (*naïve*). In a *Community History Experiment*, offspring of *selected* plants were planted in three test environments: their original plant communities with old soil (of the long‐term Jena Experiment), newly assembled plant communities with old soil, and newly assembled plant communities with new soil. In a *Selection Experiment*, we compared *selected* plants with *naïve* plants, both grown in the *selected* plants' original environment. In all test environments, increasing species richness was associated with a decrease in plant individual biomass, reproductive output, relative growth rate, plant height, leaf greenness, and leaf nitrogen concentration, and an increase in specific leaf area (SLA). In the Selection Experiment, *selected* plants had a weaker decline in biomass, taller stature, and higher leaf carbon and nitrogen concentrations than *naïve* plants with increasing species richness. In the Community History Experiment, survival was lower, while plant height, SLA, leaf nitrogen, and carbon concentrations were highest in the test environment with new plants and soil. However, in high‐diversity communities, individuals produced more biomass, grew taller, and had higher leaf greenness in their original environment. Overall, we found that, despite the crucial role of phenotypic plasticity for trait adjustments to the actual environment, selection in the biodiversity experiment produced adaptive phenotypic responses, largely explained by plant community history and positive plant–soil feedbacks established over time.

## Graphical abstract

We conducted a transplant experiment for nine plant species in a 17‐year‐old biodiversity experiment (Jena Experiment) to disentangle the role of adaptation and phenotypic plasticity in phenotypic responses to plant diversity. Selection in the biodiversity experiment produced adaptive phenotypic responses, largely explained by plant community history and positive plant–soil feedbacks established over time.

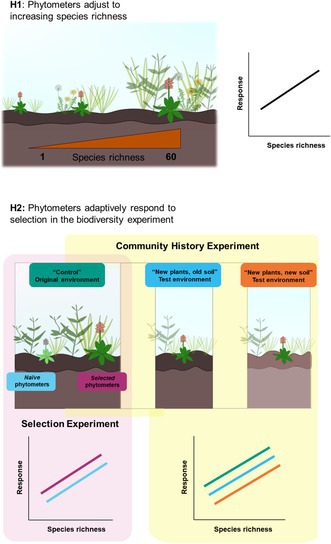

## 1 Introduction

Biodiversity is declining globally across different levels of organization: from genes to species to whole ecosystems (Cadotte et al. 2008; van der Plas 2019). Therefore, arising concerns motivated the establishment of numerous biodiversity experiments, whose major finding showed that low‐diversity communities have a lower primary productivity and are less stable than high‐diversity communities (Roscher et al. 2005; Wagg et al. 2022). However, while increasing species diversity leads to increased productivity at the community level, responses in biomass production of individual species are mixed and highly species‐specific (Hille Ris Lambers et al. 2004; Schnitzer and Bongers 2011; van Ruijven and Berendse 2003). The environments experienced by species in plant communities of varying diversity are characterized by different abiotic conditions and biotic interactions. Consequent phenotypic responses are measurable in components of plant individual performance, such as plant biomass, individual growth rate, or reproductive output. For example, due to the greater neighbor biomass and density in species‐rich communities, plants might invest more resources in vegetative growth and less in reproductive growth (Levins 1968). Indeed, previous studies found that species diversity negatively affected the proportion of flowering individuals, although the responses were highly species‐specific (Lipowsky et al. 2011; Roeder et al. 2019; Schmidtke et al. 2010). Moreover, the taller and denser canopy typical of high‐diversity communities usually induces responses to avoid shade, e.g., by increasing plant height, or to tolerate it, e.g., by increasing specific leaf area (SLA) (Bachmann et al. 2018; Lipowsky et al. 2011, 2015; Lorentzen et al. 2008).

Therefore, the same plant species growing either at high or low diversity produces phenotypic responses that can be a plastic adjustment or a result of the selection of genotypes better fitting to their selecting environment (Bailey et al. 2006; Linhart 1988; Post and Palkovacs 2009). Phenotypic plasticity involves physiological, anatomical, or morphological changes within an organism's lifetime that may improve its performance or survival in response to its actual growth environment. In turn, adaptation occurs over the time of several generations, and it requires a species to acquire or recombine genes associated with traits that improve performance or survival in its original environment (Demmig‐Adams et al. 2008).

To disentangle the roles of phenotypic plasticity and adaptation in plant populations' responses to different diversity over multiple generations, recent research has turned to long‐term biodiversity experiments. Previous studies found that plants respond phenotypically to selection in high‐ vs. low‐diversity communities, resulting in higher performance for plants selected in species‐rich communities and the opposite for plants selected in species‐poor ones (Dietrich et al. 2021; van Moorsel et al. 2018). These findings suggest an important role of plant community diversity as a selective agent. For example, in grasslands, more diverse communities are characterized by increased complementarity (Barry et al. 2019) through the diversification of the use of space and light (Lorentzen et al. 2008) or partitioning of soil resources (Fornara and Tilman 2008; Roscher et al. 2008). Another potential selective driver in plant communities of different diversity is the interaction of plant species with biotic and abiotic soil conditions, i.e., plant–soil feedback. For example, previous studies showed that plant functional groups respond to their associated soil community differently: while legumes seemed to be not affected, small and tall herbs, respectively, experienced a negative and positive plant–soil feedback, caused by an interaction with their own soil biota (Cortois et al. 2016). Focusing on the diversity of a plant community, monocultures of most species experience a decline in productivity over time (Dietrich et al. 2020), attributable to a negative plant–soil feedback caused by the accumulation of host‐specific pathogens (Schnitzer et al. 2011; Thakur et al. 2021). Conversely, at high diversity, positive plant–soil feedbacks are common (Eisenhauer et al. 2012; van Ruijven et al. 2020). In grassland biodiversity experiments, the eco‐evolutionary importance of plant–soil feedbacks can be disentangled thanks to the short generation time of both soil organisms and many plant species (Roeder et al. 2017; TerHorst and Zee 2016). However, direct evidence on how plant diversity and soil organisms produce phenotypic responses of plant species, and to which extent they may be plastic adjustments or adaptations, is still lacking. For this purpose, we established a transplant experiment to compare performance and trait expression of phytometers—test plants used to “measure” their environment via survival, growth, and reproduction (Clements and Goldsmith 1924; Strobl et al. 2018)—which experienced or did not experience the selective pressures of a grassland biodiversity experiment (Jena Experiment; Roscher et al. 2004). Taking advantage of the long‐term experiment, we aimed to separate the role of plasticity and adaptation in producing phenotypic responses. Additionally, to understand how these phenotypic responses are related to plant and soil community history, we used the so‐called ΔBEF Experiment, previously established in the Jena Experiment (Vogel et al. 2019), to create test environments with different combinations of plant and soil community histories. We hypothesized that: (1) phytometers in communities of different diversity respond to the different actual environments experienced during their growth. These responses are measurable as modifications in individual performance and phenotypic adjustments to increasing species diversity. (2) Selection in different communities of the biodiversity experiment produced adaptive responses to their original environments in plants. These responses are measurable as higher performance and a positive response to increasing species diversity when plants are grown in their original environment.

To test our second hypothesis, we studied the effects of selective pressures from two different perspectives. First, we focused on the influences of test environments with different community histories on the phytometers phenotypic responses (Section 2.2.1). One of the test environments (“control”) corresponded to the original environment in which the ancestors of the phytometers had been selected for 17 years. The other two test environments were either composed of newly assembled plant communities with soil from the Jena Experiment (new plants, old soil) or newly assembled plant communities with new soil (new plants, new soil). Second, we focused on the effects of selection history on the phenotypic responses of phytometers (Section 2.2.2). For this purpose, we again used *selected* phytometers, whose ancestors underwent the selective pressures of the original environment (“control”) and transplanted them into the same original environment. At the same time, *naïve* phytometers, whose ancestors never experienced selective pressures in the biodiversity experiment, were transplanted into the same original environment.

## 2 Materials and Methods

### 2.1 Experimental Design of the Jena Experiment

The study was conducted in the Jena Experiment, a long‐term biodiversity experiment located in the floodplain of the Saale River near Jena (Thuringia, Germany, 50°55′ N, 11°35′ E, 130 m a.s.l.) (Roscher et al. 2004). The experiment was established in 2002 on a former fertilized arable field. The soil on the site is an Eutric Fluvisol (ISRIC 1997), whose texture varies from sandy loam to silty clay with increasing distance from the river. The experiment was organized in four blocks parallel to the riverside according to these soil characteristics (Roscher et al. 2004). A pool of 60 grassland species typically growing in meadows of the *Arrhenatherion* type (Ellenberg 1988) was chosen for the experiment and classified into four functional groups: 16 grass species, 12 small herb species, 20 tall herb species, and 12 legume species. Species classification into functional groups was based on a multivariate analysis of morphological, phenological, and physiological traits compiled from the literature (for details see Roscher et al. 2004). The large species pool enabled the creation of different species mixtures, employing a near‐orthogonal design to cross the experimental factors species richness (ranging from 1 to 60, including levels 1, 2, 4, 8, 16, and 60) and functional group number (1, 2, 3, or 4). In 82 plots of the main experiment, each species‐richness level was represented with 16 replicates of different species compositions, with the exception of the 16‐species mixtures, which had only 14 replicates, and the 60‐species mixture with one unique species composition but four replicates. Species for the different mixtures were randomly chosen from the respective functional groups. For the initial sowing of the experiment in May 2002, sowing density was 1000 viable seeds per m^2^, equally distributed among species in the mixtures. The seeds were purchased from a commercial supplier specialized in seeds of regional provenance (Rieger‐Hofmann GmbH, Blaufelden‐Raboldshausen, Germany). Some poorly established species were re‐sown in autumn 2002 (for details see Roscher et al. 2004). Plots were mown twice per year (in early June and September) and the mown material was removed. Weeding was generally carried out two to three times per year to maintain the intended species combinations. The experiment was not fertilized.

In this study, we used the ΔBEF Experiment (= Determinants of Long‐Term Biodiversity Effects on Ecosystem Functioning) which was started in 2016 (Vogel et al. 2019). For the ΔBEF Experiment three subplots of 1.5 × 3 m size were established on each of the main experimental plots. These subplots correspond to three test environments varying in soil history and plant community history. The first test environment, “control”, was a subplot of the main experimental plot where communities were established in 2002. For the second test environment, the plant sod was removed with a digger, while the soil was mixed and homogenized to a depth of 30 cm on the respective subplot to obtain the combination “new plants, old soil”. The third test environment, “new plants, new soil”, was obtained by removing the vegetation and excavating the soil to 30 cm depth, which was replaced by soil from an adjacent arable field. Similarly to the old soil in 2002, the new soil had a high level of fertilization and it was used for cultivation of vegetables and cereals before. Therefore, the properties of the new soil were comparable to the properties of the old soil when the Jena Experiment was established (Vogel et al. 2019). For the second and third test environment, plastic sheets were installed as soil barriers in the top 30 cm of the soil to prevent a lateral mixing of the soils. Furthermore, for these two treatments plot‐specific plant mixtures were sown in early May 2016, with a total density of 1000 viable seeds m^−2^ equally divided among species using seeds purchased from the same supplier as for the initial establishment of the Jena Experiment in 2002. Initial measurements after the establishment of the ΔBEF Experiment showed, microbial biomass and nematode abundance were highest in communities in the “control” with shared soil and plant history and lowest in communities with “new plants, new soil” (Vogel et al. 2019). Communities with old or new soil did not differ in the number of established plant species after sowing. After the establishment of the communities, aboveground biomass production increased with species richness in all test environments, but the strength of species‐richness effects was greatest in the “control” (Vogel et al. 2019).

### 2.2 Study Species

A subset of nine species representing the four functional groups of the Jena Experiment was chosen for the present phytometer experiments. Previous research in the early years of the Jena Experiment showed that species belonging to different functional groups differ in their trait values while showing similar phenotypic responses to increasing species richness (Roscher et al. 2018). However, knowledge about whether these phenotypic responses are a plastic adjustment or an adaptation to long‐term selective pressures and how generalizable that is across species belonging to different functional groups is still lacking. Therefore, the criteria for choice were: representing the four functional groups, a good representation of the species along the plant diversity gradient, and the feasibility of collecting the required number of seed families from each community. The functional group “tall herbs” was represented by the species *Geranium pratense* L. (Geraniaceae), *Ranunculus acris* L. (Ranunculaceae) and *Crepis biennis* L. (Asteraceae), while “small herbs” were represented by *Plantago lanceolata* L. and *Plantago media* L. (both Plantaginaceae). *Lotus corniculatus* L. and *Medicago × varia* Martyn (both Fabaceae) represented the “legumes”, and *Alopecurus pratensis* L. and *Trisetum flavescens* (L.) P. Beauv. (both Poaceae) represented the “grasses”. *Crepis biennis* is monocarpic biennial to perennial, while the other eight species are perennials. To avoid the sampling of clonal replicates, we chose species whose individual genets were distinguishable for a long time after germination.

#### 2.2.1 Community History Experiment

**FIGURE 1:**
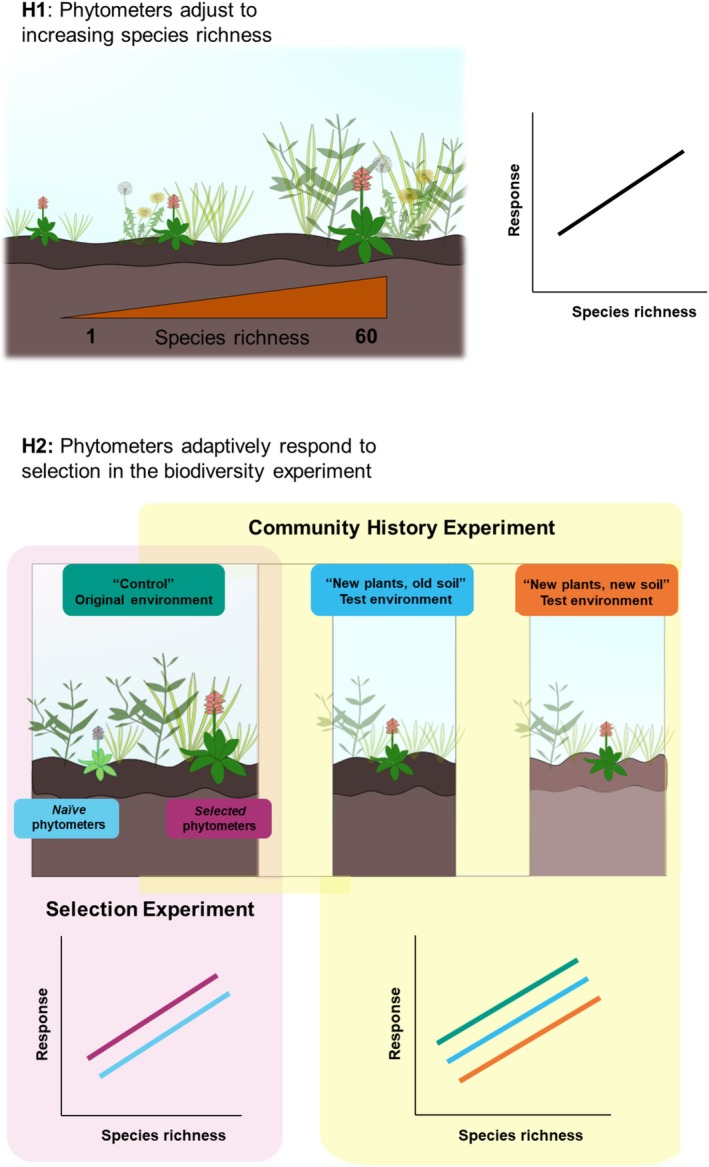
Overview of the experimental design to test our two hypotheses. For the first hypothesis (H1), we compared the response (measured as performance and trait expression) of phytometers grown in plots of the Jena Experiment to species richness. For the second hypothesis, we tested adaptive responses of phytometers in the plant communities of different diversity using two approaches. With the Selection Experiment, we compared performance and trait expression of *selected* vs. *naïve* phytometers. *Selected* phytometers were planted in their original environment “control” in the same communities where their ancestors have been selected for 17 years. *Naïve* phytometers, which never experienced selection in the biodiversity experiment, were also planted in the original environment of *selected* ones. With the Community History experiment, we compared the performance and trait expression of *selected* phytometers planted in their original environment “control”, in newly established plant communities with old soil “new plants, old soil” and in newly established plant communities with new soil “new plants, new soil”. The illustration was partly made using Inkscape.

The aim of the Community History Experiment was to test adaptive responses of selected phytometers by comparing their performance in their original environment and in test environments differing in plant and soil community history (Figure 1). For each phytometer species, 6–12 plots of different species richness, where the species belonged to the sown species combinations, were chosen. Our choice was limited by space, i.e. a maximum of two phytometer species could be planted per subplot of the ΔBEF experiment. However, for each species we aimed to include as many plots as possible, thus resulting in a dataset composed of nine species, distributed on a total of 54 plots. Out of the 54 plots, 23 of them hosted plants of two phytometer species (Table S2). Seeds of the phytometer species were collected from four mother individuals in each plot (thus creating four seed families) in the original environment (control) in 2019. The seeds were cleaned and stored at room temperature until the start of the phytometer experiments. In early January 2020, two to three seeds were placed in QuickPot trays with cells of 183 cm^3^ volume (Herrmann Meyer KG, Rellingen, Germany), which contained autoclaved soil (twice for 40 min at 121°C) from the field site mixed with sterile mineral sand (25 vol%). If more than one seedling germinated in a cell, these were removed to allow the growth of single plantlets. To break dormancy and promote germination, seeds of *L. corniculatus* were scarified and seeds *R. acris* were treated with a solution of gibberellic acid (1000 mg L^−1^ for 24 h) (Roscher et al. 2004). Afterwards, the pre‐treated seeds of *R. acris* were germinated in petri dishes on moist filter paper, and the seedlings were transferred to the QuickPots when the radicle was visible. Pre‐germination in petri dishes was also used for some seed families with low germination rates in other species to get enough seedlings. The phytometer plants were cultivated in a greenhouse (experimental field station Bad Lauchstädt, Saxony‐Anhalt, Germany) at 18°C during the day (14 h) and 12°C during the night (8 h). In mid‐March 2020, the trays were placed in a greenhouse with outside temperature and light conditions in order to harden the plants before being planted in the field. Between 4 and 15 April 2020 (see Table S2 for more details), three offspring per seed family were planted into the same community of the original environment where their seeds had been collected, which corresponded to the ΔBEF treatment “control”. Three further offspring per seed family were transplanted into the test environments “new plants, old soil” and “new plants, new soil”, respectively. In total, 36 plants (= 3 test environments × 4 seed families × 3 offspring) were planted per chosen plot and species. In a few cases, the number of seed families or offspring per seed family was not sufficient to get three offspring per subplot. In these cases the phytometer plants were distributed as equally as possible among the test environments. Planting distance was 20 cm with 25 cm distance from the plot margins; offspring of the seed families were randomly assigned to the planting positions. After planting, the phytometer plants were watered every second day for four weeks to assure a successful establishment. *Crepis biennis* phytometers were planted in the field in autumn (2 October 2020) in the same way with the germination and growth of the phytometers in the greenhouse starting in July 2020. The design resulted in a total of 2585 planted phytometers.

#### 2.2.2 Selection Experiment

The aim of the Selection Experiment was to compare the performance of phytometers whose ancestors experienced either the original environment in the biodiversity experiment (= *selected*) or not (= *naïve*) (Figure 1). The selected phytometers were the same individuals used for the Community History Experiment in the original environment “control”, i.e., their original environments were the old communities sharing plant and soil history since 2002. The second group, composed of *naïve* phytometers, was obtained by growing plants from the initial seed lots used to establish the Jena Experiment in 2002. The ancestors of the *naïve* phytometers were germinated in summer 2018 from seeds that had been stored at −20°C since 2002. Seedlings were grown in a greenhouse with a standard substrate and planted outside in autumn in a planting bed at the Experimental Field Station Bad Lauchstädt. Seeds of these individuals were also collected in summer 2019. Their seedlings were grown and planted as *naïve* phytometers in the same original environment “control” of the *selected* phytometers, as described above for the Community History Experiment. This time, three offspring of the same four seed families per species (=12 phytometers per plot × species combination) were planted in all plots, while seed families of the *selected* phytometers were plot‐specific. In total, we planted 936 *naïve* phytometers, to be compared with 884 *selected* phytometers.

### 2.3 Sampling and Measurements

Measurements of phenotypic traits were taken simultaneously for both experiments. Before planting the phytometers in the field in April 2020, initial plant height was measured as the stretched shoot length or length of the longest leaf (in case of rosette plants). The number of leaves or shoots was counted, according to the morphology of the species (= time point t0). Between 18 August and 7 September 2020, about four months after planting in the field (= time point t1), we recorded the survival rate, considering a plant as alive when at least one green leaf could still be found. Then, stretched plant height was measured and the number of leaves was counted following the methods described above. Leaf greenness, an estimate of leaf chlorophyll concentrations, was assessed by measuring the absorption of two different wavelengths (650 and 940 nm) with a portable chlorophyll meter (SPAD 502 Plus, Konica Minolta). Data were recorded for each individual by two averaged readings on different fully expanded leaves. Between 17 and 24 May 2021 (= time point t2), survival and plant height were recorded again. Afterwards, between 25 May and 10 June, one to five fully developed leaves per individual (depending on leaf and plant individual size) were sampled and stored in plastic bags in a cooling box until further processing. Leaf area was measured with a leaf area meter (LI‐3000C Portable Leaf Area Meter equipped with LI3050C transparent belt conveyor accessory, LI‐COR, USA) and samples were weighed after drying at 70°C for 48 h. Specific leaf area (SLA, ${\mathrm{mm}}_{\text{leaf}}^{2}\;{\mathrm{mg}}_{\mathrm{dw}}^{-1}$) was calculated as the ratio between leaf area and dry mass. To determine nitrogen and carbon concentrations, leaves of the individuals composing a seed family for each subplot were pooled and milled to a fine powder with a ball mill (MM200, Retsch, Haan, Germany). Sub‐samples of the milled material were scanned with an MPA FT‐NIR spectrometer (Bruker, Bremen, Germany) to determine nitrogen ($\mathrm{mg}\;N\;g_{\mathrm{dw}}^{-1}$) and carbon concentration ($\mathrm{mg}\;C\;g_{\mathrm{dw}}^{-1}$). To establish and validate the optimal NIRS model for predicting N (N_Leaf_) and C (C_Leaf_) concentrations, around 20 samples per species were processed by conventional chemical analysis using an elemental analyzer (Vario EL cube, Elementar Analysensysteme GmbH, Langenselbold, Germany). The NIRS calibration models used to predict N and C concentration in the samples had coefficients of determination *R* ^2^ = 0.986 for N and *R* ^2^ = 0.827 for C. The final trait measurements and biomass harvest took place between 18 August and 9 September 2021 (= time point t3), in order to have repeated measurements comparable across the years. First, survival was assessed, and then the plant individuals were harvested at the soil surface. Plants were stored in separate plastic bags in a cooling box until measurements took place in the laboratory. There, stretched plant height was measured, leaf or shoot number was counted, and leaf greenness was recorded as above. Afterwards, plants were dried at 70°C for 48 h and weighed to get plant individual biomass. During both the field campaigns in spring and summer 2021, the presence of inflorescences for each individual was noted. Combining these two time points to account for the different species‐specific flowering times during the growing season, we obtained an estimate of the individual reproductive status for the year 2021. Using the repeated measurements of leaf or shoot number taken during the first measurements in the field and the final harvest, we calculated relative growth rates for each individual as

(1) $$\mathrm{RGR}=\left(\ln\;{\mathrm{no}}_{t\_\text{x3}}–\ln\;{\mathrm{no}}_{t\_\text{x1}}\right)/d$$

where, ln no_t_x3_ and ln no_t_x1_ represent the natural logarithms of the number of leaves or shoots counted at the end of the experiment in August–September 2021 (t3) and in August–September 2020 (t1), and d is the number of days between these time points (Harper 1977). During all field campaigns, the height of the surrounding vegetation around the phytometers was measured in each subplot, as the average of three measurements across the area occupied by the experimental plants. For variables derived from the measurements, see Table S1.

### 2.4 Data Analyses

Data analysis was performed with R Statistical Software (v4.2.2; R Core Team 2022, http://www.R‐project.org). To test our hypotheses, we performed analyses of variance. For the Community History Experiment, we optimized a model for all the response variables fitting different explanatory variables in the following order: block, sown species richness (log‐linear), test environment of the ΔBEF experiment (factor with three levels) and its interaction with sown species richness, followed by the functional group identity of the phytometer species (factor with four levels) and its interaction with sown species richness and test environment, species identity of the phytometer species (factor with nine levels, when fitted after functional groups measuring variation within these among species) and its interaction with sown species richness and test environment, plot and its interaction with species identity, subplot and its interaction with species identity, and finally seed family identity and its interaction with test environment. Because of the nested experimental design, we calculated *F* and *p* values using the appropriate error terms as in mixed models (for details, see Table S3). To investigate the role of test environment in determining the height of the surrounding vegetation of each plot, we created a model fitting block, sown species richness (log‐linear), test environment and its interaction with sown species richness, followed by plot and subplot identity. Differences among the test environments and the four functional groups were identified using Tukey's HSD test (R package *multcomp*) (Hothorn et al. 2008). For the Selection Experiment, we adjusted the previous model by substituting the term test environment as well as its interactions with a term for selection history, and by removing the term subplot together with its interactions, because the Selection Experiment only used data from the original environment “control” Again, we calculated *F* and *p* values with the appropriate error terms as in mixed models (Table S4). For the analyses of binomial variables (i.e., survival and reproductive status) in both experiments, we used generalized linear mixed‐effects models with a binomial error distribution, implemented with the g*lmer* function in the *lme4* package (Bates et al. 2015). As random terms composing the null model, we fitted plot, seed family, interaction between plot and species identity, subplot, interaction between subplot and species identity, and interaction between test environment and seed family. Starting from the null model, fixed effects were added stepwise, including block, species richness (log‐linear), test environment, and interaction between test environment and species richness. Finally, we created for the analyses of both experiments an alternative model in order to account for possible effects of light competition by the surrounding vegetation that could partly explain the effects of species richness. In these models, the canopy height of the surrounding vegetation was fitted as a covariate right after the block term. When needed, the variables were log‐transformed to meet the requirements of normality.

## 3 Results

### 3.1 Effects of Species Richness, Functional Groups, and Seed Families on Plant Performance and Trait Expression

**FIGURE 2:**
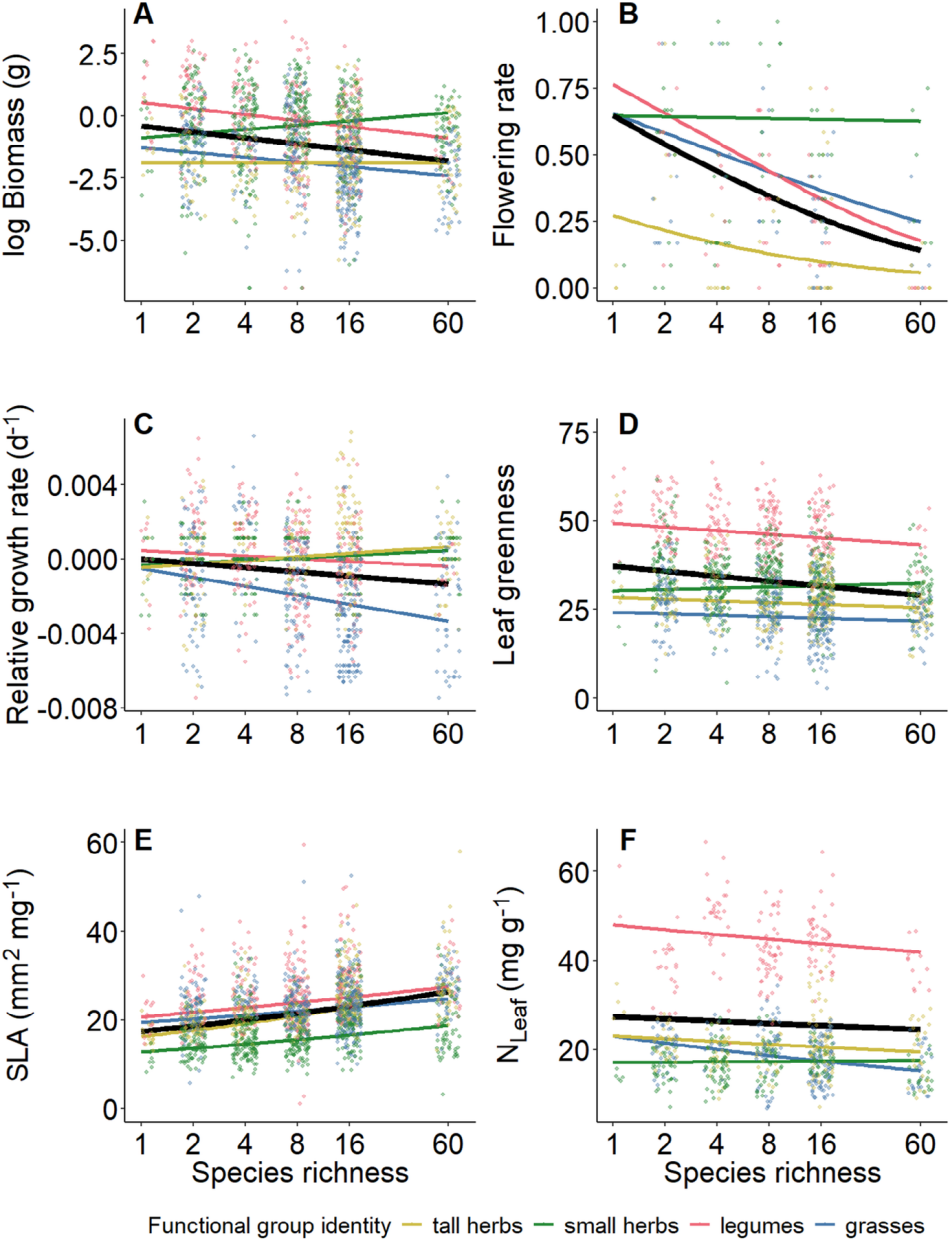
Effects of sown species richness on (A) plant individual biomass, (B) proportion of individuals reaching reproductive stage at the plot level, (C) relative growth rate (RGR) from the first to the second year, (D) leaf greenness, (E) specific leaf area (SLA), and (F) leaf nitrogen concentration (N_Leaf_). All variables were measured in the growing season after planting the phytometers. Solid colored lines represent a significant relationship at the functional‐group level, while the solid black line represents the mean response across all species.

**FIGURE 3:**
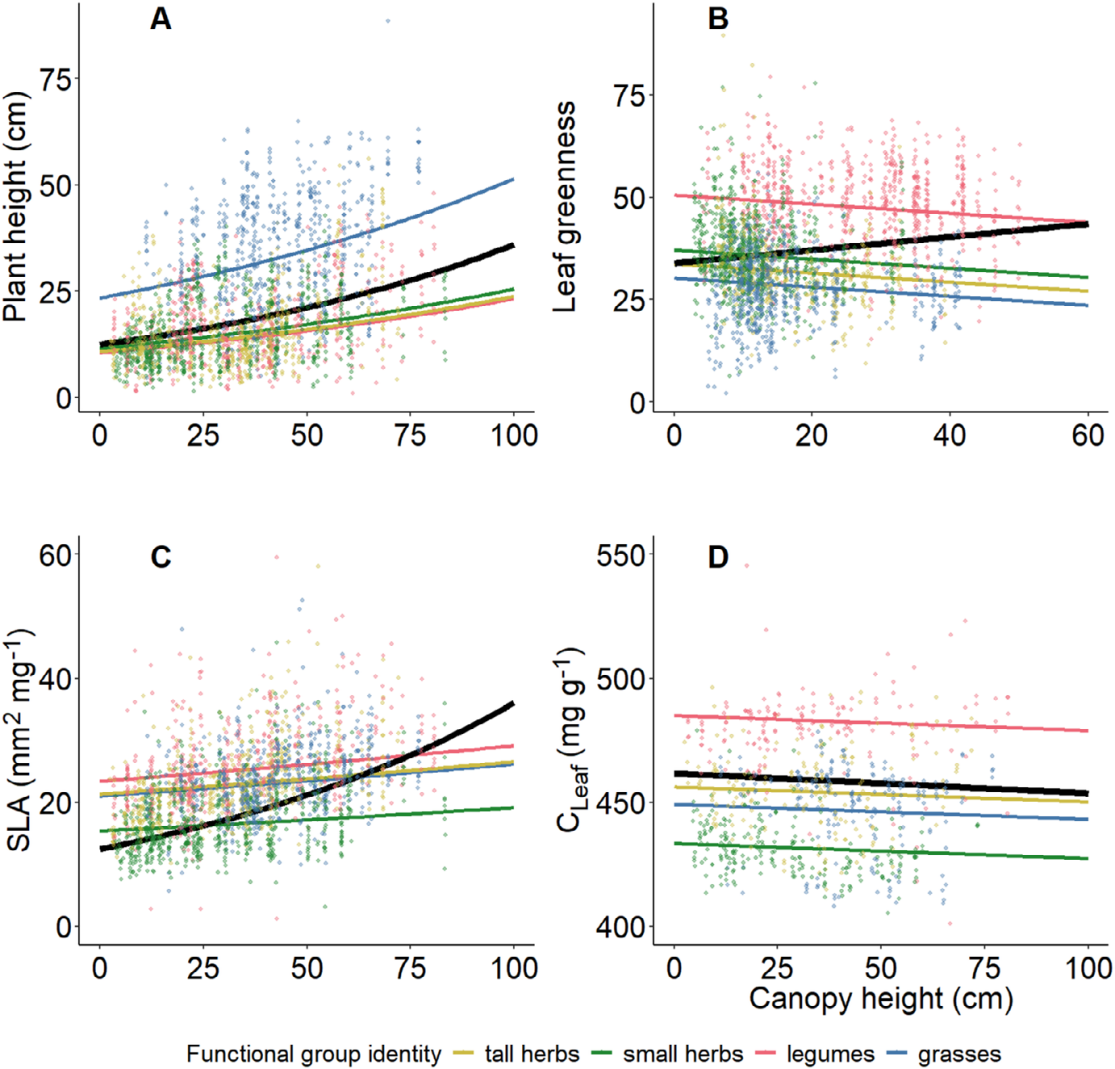
Effects of canopy height on (A) plant height, (B) leaf greenness, (C) specific leaf area (SLA), and (D) leaf carbon concentration (C_Leaf_). All variables were measured in the growing season after planting the phytometers. Solid colored lines represent a significant relationship at the functional‐group level, while the solid black line represents the mean response across all species.

Sown species richness affected plant performance and the expression of most measured traits. Specifically, plant performance measured as plant individual biomass, the proportion of plants reaching the reproductive stage, and relative growth rate (RGR) decreased with increasing species richness, while individual survival was not affected (Tables 1 and 2, Figure 2A–C and Figure S2A,B). Regarding traits related to light acquisition, plant height measured in the first year and leaf greenness measured in both years decreased, while SLA increased with species richness (Figure 2D,E and Figures S1A,B, S2C,D, S3). Plant height, measured in the second year, did not significantly respond to species richness. However, after accounting for the canopy height of the surrounding vegetation before entering species richness into the model, species richness had a significant effect on plant height in 2021 (Tables S5 and S6, Figure 3). For leaf elemental concentrations, C_Leaf_ showed little variation along the species‐richness gradient, while N_Leaf_ decreased with increasing species richness (Figure 2F and Figure S2E,F).

**TABLE 1:** Results of linear models and generalized linear mixed‐effects models for the *Community History Experiment* testing effects of sown species richness (SR), treatment, their interaction, identity of the functional group (FG‐ID), its interaction with species richness and treatment, species and its interaction with species richness and treatment on plant performance and trait expression.

Source of variation	Survival_t1		Survival_t2		Survival_t3		Reproductive status		Biomass	
	*χ* ^2^	*p*	*χ* ^2^	*p*	*χ* ^2^	*p*	*χ* ^2^	*p*	*F*	*p*
Block	7.62	0.055	13.48	0.004	11.96	0.008	0.11	0.991	5.5	0.003
Species richness (SR)	3.4	0.065	0.37	0.545	0.48	0.49	10.57	0.001	25.76	< 0.001
Treatment	3.18	0.204	12.57	0.002	12.62	0.002	0.22	0.894	2.01	0.139
SR × Treatment	0.72	0.698	3.63	0.163	3.83	0.147	2.86	0.239	2.64	0.076
FGID	12.47	0.006	6.76	0.08	10.8	0.013	22.42	< 0.001	21.34	< 0.001
SR × FGID	10.13	0.018	14.28	0.003	13.87	0.003	2.29	0.515	5.61	0.023
Treatment × FGID	10.88	0.092	5	0.544	9.18	0.164	0.68	0.995	0.89	0.544
Species‐ID	9.73	0.045	13.31	0.021	21.35	0.001	70.17	< 0.001	9.31	0.003
SR × Species‐ID	12.24	0.016	3.8	0.578	4.31	0.506	14.01	0.016	4.55	0.029
Treatment × Species‐ID	11.42	0.179	19.9	0.03	16.29	0.092	19.37	0.036	0.35	0.964
Plot	—	—	—	—	—	—	—	—	2.5	< 0.001
Species‐ID × Plot	—	—	—	—	—	—	—	—	1.22	0.292
Subplot	—	—	—	—	—	—	—	—	2.27	0.007
Species‐ID × Subplot	—	—	—	—	—	—	—	—	1.75	0.013
SF	—	—	—	—	—	—	—	—	1.66	< 0.001
Treatment × SF	—	—	—	—	—	—	—	—	0.66	1
**Source of variation**	**RGR_t3‐t1**		**Plant height_t1**		**Plant height_t2**		**Plant height_t3**			
	***F***	***p***	***F***	***p***	***F***	***p***	***F***	***p***		
Block	7.31	0.001	7.29	< 0.001	13.3	< 0.001	19.88	< 0.001		
Species richness (SR)	7.31	0.01	5.6	0.022	3.2	0.08	1.01	0.319		
Treatment	0.07	0.933	0.99	0.376	14.42	< 0.001	2.09	0.129		
SR × Treatment	2.21	0.116	1.71	0.187	1.39	0.254	3.71	0.028		
FG‐ID	18.42	< 0.001	29.48	< 0.001	75.72	< 0.001	49.81	< 0.001		
SR × FG‐ID	7.83	0.025	9.35	0.011	2.47	0.137	8.92	0.006		
Treatment × FGID	0.15	0.982	0.83	0.589	1	0.485	0.08	0.997		
Species‐ID	21.04	0.003	25.89	0.001	40.48	< 0.001	16.25	0.001		
SR × Species‐ID	1.58	0.304	7.73	0.015	3.32	0.064	1.57	0.272		
Treatment × Species‐ID	0.6	0.732	0.85	0.558	2.14	0.028	1.09	0.377		
Plot	2.11	0.002	2.92	< 0.001	3.29	< 0.001	2.97	< 0.001		
Species‐ID × Plot	1.1	0.365	1.38	0.231	1.39	0.211	1.51	0.164		
Subplot	2.21	0.029	0.91	0.643	1.03	0.484	1.92	0.026		
Species‐ID × Subplot	0.81	0.689	3	< 0.001	2.11	0.001	1.46	0.067		
SF	0.9	0.761	1.15	0.13	1.11	0.187	1.07	0.285		
Treatment × SF	1.08	0.237	1.03	0.366	1.44	< 0.001	0.96	0.677		
**Source of variation**	**LeafG_t1**		**LeafG_t3**		**SLA**		**N**_**Leaf**_		**C**_**Leaf**_	
	***F***	***p***	***F***	***p***	***F***	***p***	***F***	***p***	***F***	***p***
Block	3.46	0.024	1.21	0.315	10.26	< 0.001	31.85	< 0.001	8.3	< 0.001
Species richness (SR)	15.51	< 0.001	45.51	< 0.001	62.57	< 0.001	12.25	0.001	< 0.01	1
Treatment	1.56	0.215	0.42	0.656	8.68	< 0.001	3.59	0.031	3.15	0.047
SR × Treatment	0.41	0.666	4.38	0.015	0.29	0.747	2.4	0.096	0.26	0.769
FGID	90.4	< 0.001	121.65	< 0.001	67.77	< 0.001	206.41	< 0.001	70.91	< 0.001
SR × FGID	11.38	0.007	1	0.441	1.25	0.356	2.67	0.119	2	0.193
Treatment × FGID	2.14	0.189	0.39	0.863	0.33	0.903	0	1	0.67	0.68
Species‐ID	12.21	0.005	7.91	0.006	17.15	< 0.001	29.73	< 0.001	12.57	0.001
SR × Species‐ID	6.22	0.025	1.27	0.361	1.14	0.415	1.33	0.341	1.03	0.461
Treatment × Species‐ID	2.87	0.007	1.04	0.417	1.64	0.106	0.72	0.705	1.1	0.367
Plot	3.65	< 0.001	3.45	< 0.001	2.96	< 0.001	3.91	< 0.001	2.57	< 0.001
Species‐ID × Plot	0.81	0.564	2.03	0.05	3.41	0.002	3.59	0.001	1.84	0.078
Subplot	1.96	0.028	0.96	0.57	1.38	0.164	1.06	0.444	0.62	0.956
Species‐ID × Subplot	0.95	0.54	1.55	0.046	1.14	0.285	1.68	0.017	1.64	0.022
SF	0.9	0.802	0.99	0.518	1.02	0.42	—	—	—	—
Treatment × SF	1.07	0.199	1.16	0.056	0.95	0.729	—	—	—	—

*Note:* If variables were measured at different time points, it is indicated with t1 = summer 2020 and t3 = summer 2021. Shown are *F* (for linear models), *χ* ^2^ (for generalized linear mixed‐effects models) and *p* values. Abbreviations of variable names are C_Leaf_, leaf carbon concentration; LeafG, leaf greenness; N_Leaf_, leaf nitrogen concentration; SLA, specific leaf area. *χ* ^2^ values of binomial variables (survival rate and reproductive status) were obtained using generalized mixed effects models. For other variables, *F* values were obtained with an analysis of variance. Because of the nested design of the experiment, *F* and *p* values were calculated using the appropriate error term, which can be found in Table S3.

**TABLE 2:** Results of linear models and generalized linear mixed effects models for the *Selection Experiment* testing effects of sown species richness (SR), selection history, functional group identity (FG‐ID), species identity, and their interactions with species richness and selection history on plant performance and trait expression.

Source of variation	Survival_t1		Survival_t2		Survival_t3		Reproductive status		Biomass	
	*χ* ^2^	*p*	*χ* ^2^	*p*	*χ* ^2^	*p*	*χ* ^2^	*p*	*F*	*p*
Block	3.62	0.305	9.67	0.022	9.23	0.026	1.4	0.707	3.9	0.014
Species richness (SR)	2.17	0.14	1.42	0.234	1.49	0.223	6.64	0.01	9.57	0.003
Selection	2.12	0.145	0.37	0.545	1.12	0.289	< 0.01	0.956	28.33	0.003
SR × Selection	0.01	0.941	0.98	0.321	0	0.984	1.37	0.241	2.11	0.153
FG‐ID	6.14	0.105	2.19	0.535	13.57	0.004	14.91	0.002	14.46	< 0.001
SR × FG‐ID	4.66	0.199	10	0.019	7.5	0.058	5.92	0.116	5.08	0.035
Selection × FG‐ID	4.03	0.258	2.15	0.542	0.25	0.969	3.87	0.276	0.82	0.521
Species‐ID	4.69	0.321	8.05	0.154	21.61	0.001	57.82	< 0.001	10.79	0.003
SR × Species‐ID	4.45	0.349	5.61	0.346	3.85	0.571	10.75	0.057	7.82	0.009
Species × Selection	8.82	0.066	13.33	0.02	19.92	0.001	15.31	0.009	0.42	0.836
Plot	—	—	—	—	—	—	—	—	3.87	0.033
Species × Plot	—	—	—	—	—	—	—	—	1.69	0.113
Selection × Plot	—	—	—	—	—	—	—	—	1.65	0.009
Seed family	—	—	—	—	—	—	—	—	1.09	0.215
**Source of variation**	**RGR_t3‐t1**		**Plant height_t1**		**Plant height_t2**		**Plant height_t3**			
	***F***	***p***	***F***	***p***	***F***	***p***	***F***	***p***		
Block	5.46	0.003	5.47	0.003	8.54	< 0.001	22.18	< 0.001		
Species richness (SR)	1.58	0.215	2.45	0.125	5.16	0.028	< 0.01	> 0.999		
Selection	4.2	0.133	0.67	0.46	1.11	0.34	6.25	0.054		
SR × Selection	1.14	0.293	2.77	0.103	0.33	0.566	1.47	0.231		
FG‐ID	7.55	< 0.001	15.47	< 0.001	47.43	< 0.001	42.98	< 0.001		
SR × FG‐ID	2.08	0.245	5.48	0.037	0.64	0.61	14.39	0.002		
Selection × FG‐ID	0.42	0.751	1.13	0.409	1.23	0.362	3.89	0.063		
Species‐ID	6.28	0.054	11.35	0.006	26.98	< 0.001	48.53	< 0.001		
SR × Species‐ID	0.22	0.877	4.79	0.045	3.2	0.07	6.3	0.016		
Species‐ID × Selection	0.44	0.727	2.36	0.054	1.05	0.388	1.35	0.245		
Plot	1.26	0.463	1.9	0.215	1.74	0.207	5.61	0.011		
Species‐ID × Plot	3.15	0.016	4.07	0.001	3.65	< 0.001	1.45	0.189		
Plot × Selection	0.92	0.607	1.42	0.045	1.75	0.003	1.72	0.005		
SF	1.17	0.106	1.09	0.177	1.38	0.001	0.81	0.965		
**Source of variation**	**LeafG_t1**		**Leaf greenness_t3**		**Specific leaf area**		**N**_**Leaf**_		**C**_**Leaf**_	***p***
	***F***	***p***	***F***	***p***	***F***	***p***	***F***	***p***	***F***	
Block	1.16	0.334	1.71	0.177	7.97	< 0.001	18.57	< 0.001	5.47	0.003
Species richness (SR)	19.79	< 0.001	24.18	< 0.001	48.29	< 0.001	19.47	< 0.001	4.21	0.046
Selection	1.73	0.258	3.41	0.124	0.18	0.689	0.83	0.403	3.33	0.127
SR × Selection	1.05	0.311	0.03	0.853	1.5	0.226	4.64	0.036	4.21	0.045
FG‐ID	65.84	< 0.001	1145.75	< 0.001	54.83	< 0.001	143.2	< 0.001	44.02	< 0.001
SR × FG‐ID	5.76	0.034	1.6	0.285	1.5	0.288	5.33	0.026	0.08	0.971
Selection × FG‐ID	0.94	0.477	1.24	0.375	0.42	0.743	1.33	0.33	1.98	0.195
Species‐ID	7.88	0.014	3.6	0.075	15.57	0.001	71.2	< 0.001	9.97	0.003
SR × Species‐ID	3.68	0.076	1.14	0.431	1.26	0.368	2.8	0.094	5.39	0.018
Species‐ID × Selection	1.83	0.123	1.29	0.271	1.41	0.223	3.07	0.01	0.42	0.838
Plot	2.12	0.174	1.57	0.302	0.96	0.581	2.98	0.052	2.17	0.122
Species‐ID × Plot	2.33	0.034	1.9	0.083	4.32	< 0.001	1.28	0.254	0.76	0.64
Plot × Selection	1.4	0.052	1.12	0.297	1.09	0.333	1.1	0.301	1.03	0.424
SF	1.13	0.107	1.39	0.002	0.98	0.567	—	—	—	—

*Note:* If variables were measured at different time points, it is indicated with t1 = summer 2020 and t3 = summer 2021. Shown are *F* (for linear models), *χ* ^2^ (for generalized linear mixed‐effects models) and *p* values. Abbreviations of variable names: C_Leaf_, leaf carbon concentration; LeafG, leaf greenness; N_Leaf_, leaf nitrogen concentration; SLA, specific leaf area. *χ* ^2^ values of binomial variables (survival rate and reproductive status) were obtained using generalized mixed effect models. For other variables, *F* values were obtained with an analysis of variance. Because of the nested design of the experiment, *F* and *p* values were calculated using the appropriate error term, which can be found in Table S3.

Phytometers belonging to different functional groups differed in all measured variables with the exception of survival (Tables 1 and 2, Tables S7, S8). Moreover, species‐richness effects showed some variation among functional groups (significant interactions species richness × functional group identity). In particular, for plant individual biomass, small herbs showed a positive response along the plant‐diversity gradient, while for species of the other functional groups, plant individual biomass consistently declined with increasing species richness (Figure 2A and Figure S2A). Additionally, in spite of the general decrease in plant height with increasing species richness, both small and tall herbs increased in height (Figure S1A). Functional group‐specific responses were also detectable for leaf greenness measured in the first year, for which grasses were the only group with higher values of leaf greenness in high‐diversity communities (Figures S1B and S3A). In the Community History Experiment, RGR was positive for small and tall herbs (Figure 2C). Finally, in contrast to the general decrease in N_Leaf_ with increasing species richness, N_Leaf_ of small herbs showed the opposite response in the Selection Experiment (Figure S2E).

The nine phytometer species within functional groups differed in plant individual biomass and the expression of all traits. In contrast, the seed families used in the Community History Experiment differed in plant individual biomass, but not in the expression of the measured traits. All the seed families used for the Selection Experiment, comparing phytometers with and without selection history in the plots, differed in plant height in the first year and leaf greenness in the second year (Table 2).

### 3.2 Effects of Community History on Phytometer Performance and Trait Expression

**FIGURE 4:**
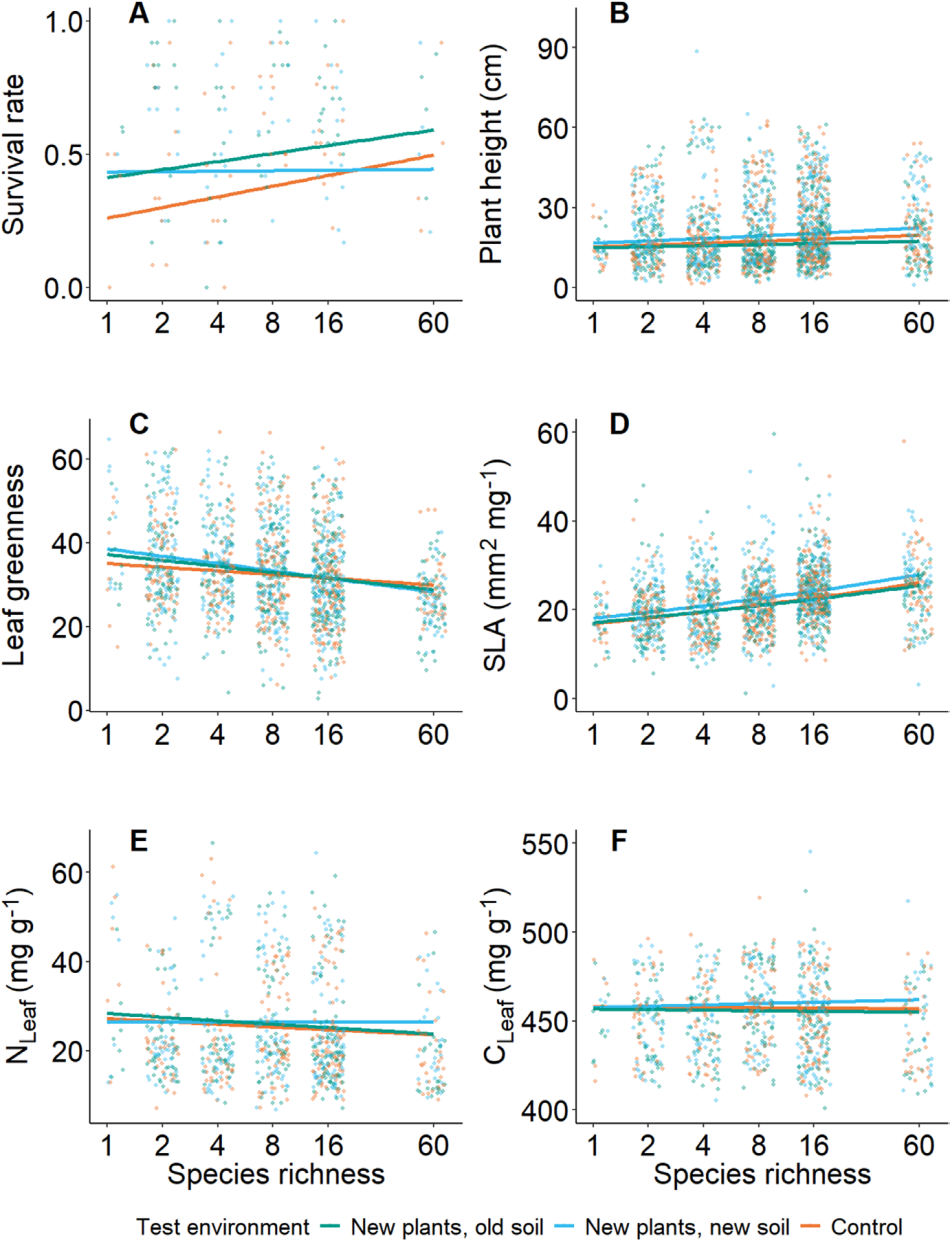
Effects of the test environments on (A) survival rate at the plot level, (B) plant height, (C) leaf greenness, (D) specific leaf area (SLA), (E) leaf nitrogen concentration (N_Leaf_), and (F) leaf carbon concentration (C_Leaf_). Solid colored lines represent a significant relationship at the treatment level. All variables were measured in the growing season after planting the phytometers. Shown are the different test environments of the ΔBEF Experiment. i.e. “control”, “new plants, old soil” and “new plants, new soil”.

**FIGURE 5:**
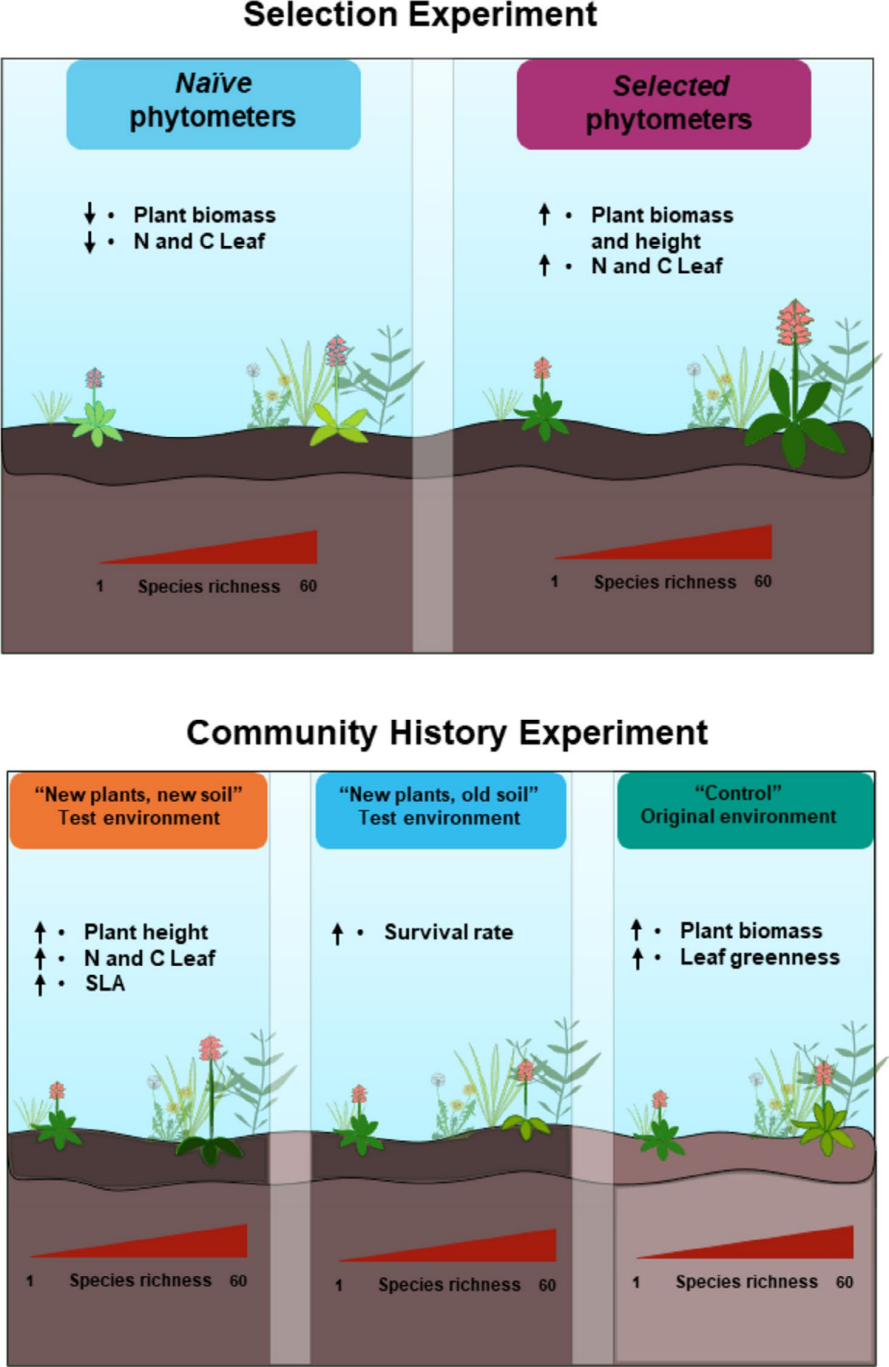
Overview of the key results tested with our second hypothesis (H2). The Selection Experiment showed that the performance of *selected* phytometers in their original environment was higher in high‐diversity communities, compared to *naïve* phytometers. The Community History experiment also showed a greater performance of phytometers in their original environment (“control”), confirming that 17 years of selection in communities of different diversity led to adaptive responses of plants, and the important role of phenotypic plasticity, visible as phenotypic responses to the test environment “new plants, new soil”. The direction of the arrows near each trait indicates the increasing or decreasing trait value along the species richness gradient. The illustration was partly made using Inkscape.

The ΔBEF test environments and its associated community histories, in which the phytometers grew, affected their survival rates, height and expression of leaf traits (SLA, N_Leaf_, C_Leaf_) (Table 1 and Table S9, Figures 4 and 5 and Figure S4). Irrespective of species richness, survival rates differed among test environments. The original environment “control” had the lowest survival rates, however, the difference was 3%–6% from the test environment “new plants, new soil” and 9%–11% from the test environment “new plants, old soil” (Table 1 and Figure 4A). Plants grew taller, had larger SLA and higher N_Leaf_ and C_Leaf_ in the test environment “new plants, new soil” (Table 1, Figure 4D,E and Figure S4B). However, when accounting for variation in canopy height, only the treatment effects on plant height remained significant (Table S5). Moreover, phytometers in the original environment “control” were shorter and had lower leaf greenness in low‐diversity communities, while those in high‐diversity communities were taller and had higher leaf greenness when compared to the other two test environments in the second summer (significant interaction between species richness × test environment; Table 1, Figures 4B and 5 and Figure S4B). When accounting for the effects of canopy height, the effects of the test environment on plant individual biomass became significant, i.e. decrease in biomass in diverse communities was less pronounced in phytometers grown in their original environment “control”, while it was more pronounced for the phytometers grown in the “new plants, new soil” environment (Table S5 and Figure S4C). Concerning the effects of the test environments on the height of the surrounding vegetation, the test environment “new plants, new soil” had the tallest canopy during spring 2021 compared to the other two test environments, while there were no significant difference in summer during both years (Table S10 and Figure S5A,C,E).

### 3.3 Effects of Selection History on Phytometer Performance and Trait Expression

**FIGURE 6:**
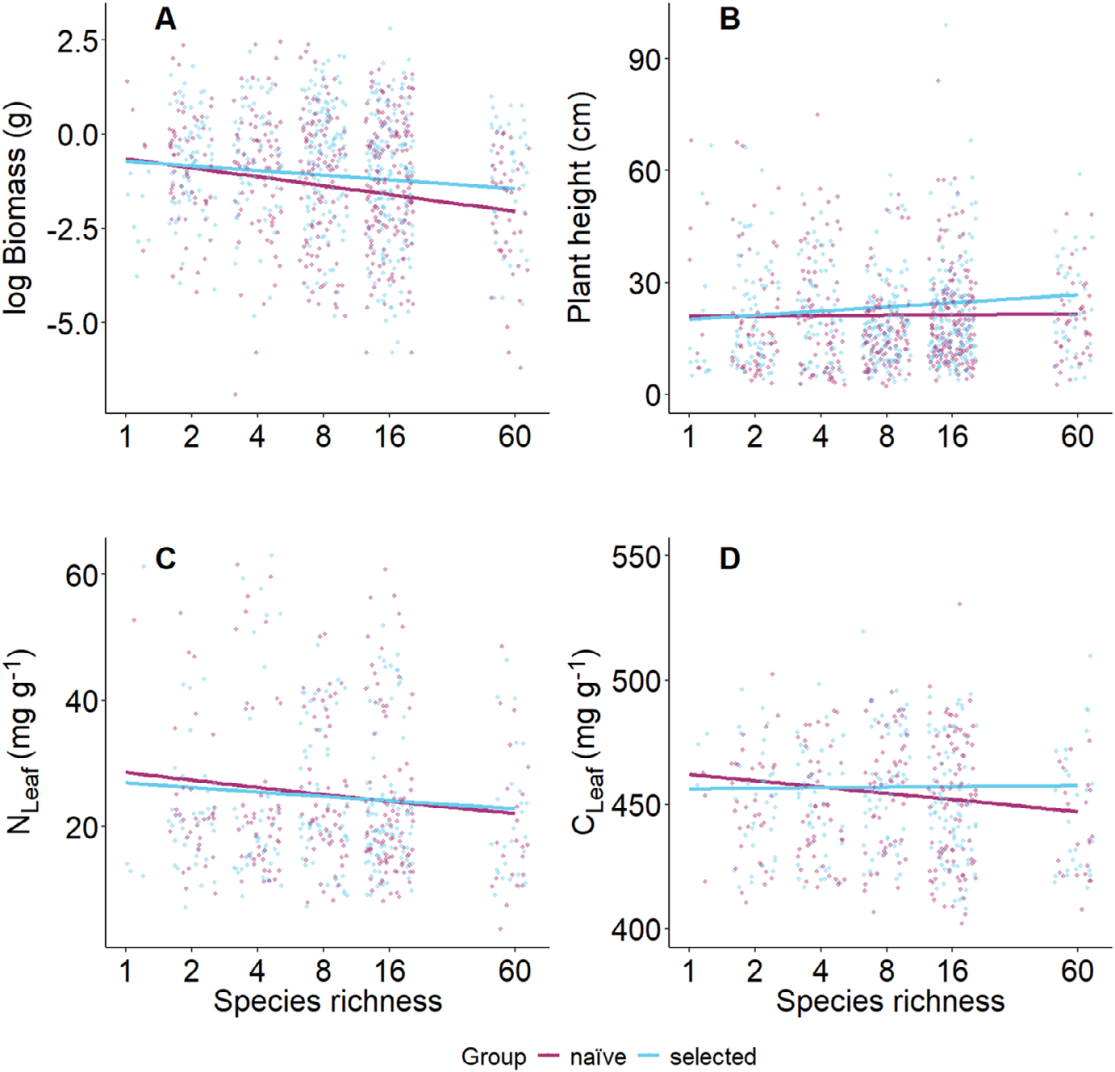
Effects of the selection history on (A) plant individual biomass, (B) plant height, (C) leaf carbon concentration (C_Leaf_) and (D) nitrogen concentration (N_Leaf_). All variables were measured in the growing season after planting the phytometers. Solid colored lines represent a significant relationship among the variables and the selection history of the phytometers.

*Selected* and *naïve* phytometers grown in the original environment “control” differed in plant individual biomass and in plant height measured in the second year (Figures 5 and 6). Specifically, *selected* phytometers grew taller and produced more biomass than the *naïve* ones. Moreover, *naïve* phytometers had higher N_Leaf_ and C_Leaf_ at low levels of species richness, while the *selected* ones had the highest nutrient concentrations at higher species richness (Table 2, Figures 5 and 6). The significant effect of selection history on plant individual biomass and plant height in the second year was mediated by the canopy height of the surrounding vegetation (Table S6).

## 4 Discussion

### 4.1 Phenotypic Responses to Plant Species Diversity and Differences Among Functional Groups (H1)

In accordance with previous studies in the Jena Experiment (summarized in Roscher et al. 2018), we found that species richness affected the expression of most of the studied traits and plant individual performance. Although survival did not change with species richness, individuals flowered less frequently, had a smaller RGR, and produced less biomass, suggesting a declining individual performance with increasing species richness. We also found effects of species richness on the expression of traits related to light and nutrient acquisition, such as lower height in the first year, increasing SLA, and decreasing leaf greenness and N_Leaf_. Confirming the role of light competition as an important driver of plant individual performance and trait expression (Bachmann et al. 2018; Thein et al. 2008), variation in plant height, as well as the effects of species richness on plant height, were largely explained by canopy height. Functional groups responded to some degree differently to increasing plant species richness. Specifically, small herbs produced more biomass, and their RGR and N_Leaf_ were higher in response to increasing species richness. Obviously, the small stature of this functional group does not allow plants to reach the upper canopy layers and acquire direct sunlight, and their adjustment to canopy shade allows them to successfully increase their performance. Morphological and physiological adjustments in trait expression may help them to cope with the highly variable supply of light during the different seasons (Roscher et al. 2011). Furthermore, the increase in RGR and N_Leaf_, as well as leaves with larger SLA, are often found in shaded plants (Valladares and Niinemets 2008). The tall herbs, on their part, responded to the increased competition for light by increasing their height and RGR, but their performance was anyway negatively affected by increasing species richness. Our results partly differ from previous findings, which showed how species that are able to grow tall enough invest their resources in increased height as well as biomass, indicating higher performance (Hirose and Werger 1995). We conclude that the actual growth environment significantly affected phytometers trait expression and performance, which generally decreased with increasing species richness. The different responses of functional groups, especially the small herbs, confirm again the mixed nature of plant diversity effects on plant performance across species.

### 4.2 Modifications of Phenotypic Responses to Selection in Communities of Different Plant Species Diversity (H2)

The main focus of our study was to test whether selection in the long‐term biodiversity experiment produces adaptive phenotypic responses to species richness. With the Selection Experiment, we tested whether *selected* phytometers performed better than *naïve* ones when both groups were grown in the *selected* phytometers' original environment. We found some evidence supporting our hypothesis regarding plant individual performance, measured as phytometer biomass and height. Irrespective of being *selected* or *naïve*, phytometers suffered from competition in highly diverse communities, resulting in smaller plants. However, this reduction in size was less pronounced in *selected* phytometers. Furthermore, we found increased C_Leaf_ and N_Leaf_ with increasing species richness in *selected* phytometers, and the opposite trend for the *naïve* ones. In summary, the performance and trait expression of the *naïve* phytometers were comparable (plant biomass and height) or better (C_Leaf_, N_Leaf_) than those of the *selected* ones in low‐diversity communities, while they were worse in high‐diversity communities (Figure 5).

Previous studies, performed on plants selected in the Jena Experiment for eight years and grown in an experimental garden with different combinations of soil biota, found that plants selected in monocultures can benefit from mutualistic interactions with the soil community over time, thus decreasing the effects of negative plant–soil feedback (Zuppinger‐Dingley et al. 2016). However, in our field study, the lower performance of selected phytometers in low‐diversity communities suggests that the effects of pathogens in the soil are, in fact, stronger because of the negative plant–soil feedback developed over time (Schnitzer et al. 2011; Thakur et al. 2021). Given the higher performance of *naïve* phytometers in low‐diversity communities, new plants transplanted into an “unknown” soil and its associated community were apparently less affected by these dynamics. High‐diversity communities are characterized by stronger competition for light and soil resources. This could explain the greater performance of *selected* phytometers and support the view that plant–soil feedbacks could vary dependent on interspecific competition (Lekberg et al. 2018). As hypothesized above, the observed advantage of *selected* phytometers in high‐diversity communities could additionally be explained by evolutionary processes, i.e., *selected* phytometers were adapted to the plant communities of their original environment and reached greater performance.

With the Community History Experiment, we tested whether performance of *selected* phytometers was higher in their original environment (“control”) compared to other two test environments, characterized by different combination of plant and soil community history i.e. “new plants, old soil” and “new plants, new soil”. After accounting for canopy height, the reduction in phytometer biomass caused by increasing species richness was less pronounced in their original environment compared to the other two test environments. These results suggest that phytometers growing in their original environment were better competitors at high diversity compared to those growing in the other two test environments (Figure 5). Positive effects of the original environment on plant performance were already found in previous studies (Johnson et al. 2010; Pregitzer et al. 2010; Wagg et al. 2015). Therefore, our findings add to previous results that more than 15 years of selection in high‐ vs. low‐diversity communities lead to local adaptation of plant phenotypes, shown both through field and previous common garden experiments (Dietrich et al. 2021; van Moorsel et al. 2018) These studies compared plants selected in monoculture vs. selection in mixtures, resulting in plants with higher performance when re‐planted in their “home‐diversity” environment than when planted into an “away‐diversity” environment (Dietrich et al. 2021; van Moorsel et al. 2018). Overall, our results show that 17 years of selection history in different biodiversity environments induced measurable plant phenotypic responses to plant and soil community diversity and history. Negative plant–soil feedbacks, typical of low‐diversity communities, significantly reduced the performance of phytometers that underwent selection in monocultures. Conversely, adaptive responses could be measured as increased plant performance and trait expression in high‐diversity communities, possibly due to adaptation to biotic environments characterized by different levels of plant species richness and associated soil communities.

However, the test environment in which phytometers grew influenced their survival rates as well as the expression of several traits, thus demonstrating an important role of phenotypic plasticity for plants responses to their actual environment (Figure 5). Contrary to our expectations, survival was generally highest in the test environment “new plants, old soil” irrespective of species richness. This contrasts our expectations based on previous findings, that showed how the presence of soil history is usually detrimental for plant performance in species‐poor communities, mostly explained by the density‐dependent accumulation of pathogens in the soil over time (Kulmatiski et al. 2012; Thakur et al. 2021). Concerning the measured traits, we found the largest effects in the test environment “new plants, new soil”. As canopy height was the tallest in this test environment, phytometers were more heavily shaded by taller plants than in the other two treatments inducing taller growth and increased SLA as a response to the shade caused by the surrounding vegetation as already suggested in previous studies of trait expression in biodiversity experiments (Bachmann et al. 2018; Lipowsky et al. 2011, 2015; Lorentzen et al. 2008). Moreover, we found higher C_Leaf_ and N_Leaf_ in the test environment “new plants, new soil”. The soil used to establish this treatment within the ΔBEF Experiment was taken from a neighboring agricultural field with similar characteristics as the soil of the field site of the Jena Experiment, when the experiment was established (Vogel et al. 2019; Wagg et al. 2022). Thus, the test environment with new soil was more fertile than the test environments with soil history, leading to higher C_Leaf_ and N_Leaf_. Moreover, values of leaf greenness were higher in the test environment “new plants, new soil”, especially at low diversity. Given that soil fertility in the test environment with new soil was probably higher, this could likely be explained by lower interspecific competition and to the more sparse vegetation, which provided favorable conditions for phytometers in monocultures to effectively exploit nutrients. In a former transplant experiment, Lipowsky et al. (2011) found that, despite the great role attributable to plastic responses, five years of selection in the biodiversity experiment already produced the first signs of local adaptation. Our experiment, performed after 17 years of selection in communities of different diversity recognized once again the importance of plasticity for phenotypic adjustments.

Our results open some prospects on possible applications for grassland restoration strategies. The importance of optimal soil conditions for ecological restoration is known (Raupp et al. 2024). Our study adds that the restoration of species‐rich grasslands could be more successful if seed material that has been selected in highly diverse grasslands is used. The positive plant–soil feedback established over time in high‐diversity communities emphasizes that conservation should prioritize the maintenance of old, species‐rich grasslands, while further research on grassland restoration should be directed on how the reestablishment of species‐rich grasslands could be improved by the transfer of seeds and soil communities (Slodowicz et al. 2023) or through microbial seed coating (Rocha et al. 2019).

## 5 Conclusions

Phytometers grown in communities of different diversity showed differentiations in performance and trait expression, confirming our hypothesis (H1) that the actual growth environment, differing in species richness, produces measurable phenotypic differences. Furthermore, in spite of the crucial role of phenotypic plasticity for trait adjustment, the greater performance and different trait expression of phytometers in their environment of origin could be observed with both our experimental approaches. Therefore, we propose that adaptive responses to original environmental conditions together with phenotypic plasticity shape plants phenotypic differentiation, partially confirming our second hypothesis (H2). To better understand co‐evolutionary processes between plant and soil communities, we encourage more field studies investigating both the direct roles of plant community history and soil community on selected vs. unselected plants.

## Author Contributions

**Francesca De Giorgi:** conceptualization (equal), data curation (lead), formal analysis (equal), investigation (lead), visualization (lead), writing – original draft (lead). **Walter Durka:** conceptualization (equal), funding acquisition (lead), project administration (lead), supervision (lead), writing – review and editing (equal). **Yuanyuan Huang:** formal analysis (lead), visualization (lead), writing – review and editing (supporting). **Bernhard Schmid:** writing – review and editing (equal). **Christiane Roscher:** conceptualization (equal), data curation (supporting), formal analysis (supporting), funding acquisition (lead), investigation (supporting), project administration (lead), supervision (lead), writing – review and editing (equal).

## Conflicts of Interest

The authors declare no conflicts of interest.

## Supporting information


Data S1.

## Data Availability

This work is based on data elaborated by the subproject10 “Plant trait variation and evolution in the biodiversity‐ecosystem functioning context” of the Jena Experiment, which is funded by the Deutsche Forschungsgemeinschaft (FOR5000). The datasets and code are publicly available in the Jena Experiment database (https://jexis.idiv.de/), (https://doi.org/10.25829/3YSY‐WS93, https://doi.org/10.25829/PWEN‐4J37).
