# Supplementary material for: Selection and Phenotypic Plasticity Shape Plant Performance in a Grassland Biodiversity Experiment
Source: Ecol Evol. 2025 Mar 13;15(3):e71117. doi: 10.1002/ece3.71117 (PMC11904805; doi:10.1002/ece3.71117)

# Supplementary Material

Table S 1: Overview of the phenotypic traits measured on phytometer plants during the course of the experiment, where t0 = initial measurement at planting in spring 2020, t1 = summer 2020, t2 = spring 2021, and t3 = summer 2023.

| Variable | Function | Unit | Description | Measured | | | |
| --- | --- | --- | --- | --- | --- | --- | --- |
|  |  |  |  | t0 | t1 | t2 | t3 |
| Survival | Performance | / | Survival of the individual |  | x | x | x |
| Reproductive status | Performance | / | Presence or absence of flowers |  |  | x | x |
| Plant biomass | Performance | g | Total aboveground biomass |  |  |  | x |
| Leaf/Shoot number | Light acquisition | / | Number of shoots or leaves | x | x |  | x |
| Plant height | Light acquisition | cm | Length of the longest shoot or leaf | x | x | x | x |
| Leaf greenness | Light acquisition | / | Estimate of chlorophyll concentration |  | x |  | x |
| Specific leaf area | Light acquisition | mm^2^_leaf_ g^-1^_leaf_ | Leaf area per leaf dry mass |  |  | x |  |
| N_Leaf_ | Nutrient acquisition | mg N g_dw_^-1^ | Leaf nitrogen concentration |  |  | x |  |
| C_Leaf_ | Nutrient acquisition | mg C g_dw_^-1^ | Leaf carbon concentration |  |  | x |  |

Table S2: Overview of the experimental design of the phytometer experiment showing the species used in the experiment, the number of plots per species-richness level in which they were transplanted and where they belong to the sown composition (in brackets), the planting dates and the number of phytometers for each species.

| Species | Species-richness level | | | | | | Planting date | No. phytometers |
| --- | --- | --- | --- | --- | --- | --- | --- | --- |
|  | 1 | 2 | 4 | 8 | 16 | 60 |  |  |
| *Geranium pratense* | 1(1) |  | 1(1) | 1(1) | 4(7) | 1(4) | 12-15/04/20 | 269 |
| *Ranunculus acris* |  | 2(2) |  | 2(2) | 3(3) | 1(4) | 09/04/20 | 287 |
| *Crepis biennis* | 1(1) |  | 2(2) |  | 2(3) | 1(4) | 02/10/20 | 256 |
| *Plantago lanceolata* | 1(1) | 2(3) | 2(5) | 2(2) | 2(3) | 1(4) | 08-09/04/20 | 567 |
| *Plantago media* |  | 1(1) | 2(2) | 3(3) | 3(4) | 1(4) | 09-10/04/20 | 432 |
| *Lotus corniculatus* |  | 1(1) | 1(1) | 4(4) | 3(4) | 1(4) | 14/04/20 | 453 |
| *Medicago x varia* | 1(1) | 1(1) | 2(2) | 1(1) | 3(3) | 1(4) | 09-14/04/20 | 393 |
| *Alopecurus pratensis* |  | 1(1) | 0(1) | 2(2) | 4(4) | 1(4) | 04-05/04/20 | 384 |
| *Trisetum flavescens* |  | 2(2) | 1(1) | 3(3) | 3(3) | 1(4) | 06-07/04/20 | 480 |

Table S3: Summary of the analysis of Community History Experiment using linear models. Shown are the degrees of freedom, mean square values and calculation of F values for each explanatory variable. Degrees of freedom (DF) refer to n = 1509 individuals, which differ according to the variable and the time of measurements.

| Source of variation | DF | Mean Square | F |
| --- | --- | --- | --- |
| Block (B) | 3 | MS_B_ | MS_B_/MS_P_ |
| Species richness (SR) log-scale | 1 | MS_SR_ | MS_SR_/MS_P_ |
| Treatment (T) | 2 | MS_T_ | MS_T_/MS_SP_ |
| SR x T | 2 | MS_SR x T_ | MS_SR x T_/MS_SP_ |
| Functional group identity (FG-ID) | 3 | MS_FG-ID_ | MS_FG-ID_/MS_P_ |
| SR x FG-ID | 3 | MS_SR x FG-ID_ | MS_SR x FG-ID_/MS_Sp-ID x P_ |
| T x FG-ID | 6 | MS_T x FG-ID_ | MS_T x FG-ID_/ MS_Sp-ID x P_ |
| Species identity (Sp-ID) | 5 | MS_Sp-ID_ | MS_Sp-ID_/ MS_Sp-ID x P_ |
| SR x Sp-ID | 5 | MS_SR x Sp-ID_ | MS_SR x Sp-ID_/ MS_Sp-ID x P_ |
| T x Sp-ID | 10 | MS_T x Sp-ID_ | MS_T x Sp-ID_/MS_SP_ |
| Plot | 47 | MS_P_ | MS_P_/MS_SP_ |
| Sp-ID x Plot | 8 | MS_Sp-ID x P_ | MS_Sp-ID x P_/MS_SP_ |
| Subplot | 100 | MS_SP_ | MS_SP_/MS_Sp-ID x SP_ |
| Sp-ID x Subplot | 28 | MS_Sp-ID x SP_ | MS_Sp-ID x SP_/ MS_T x SF_ |
| Seed family | 210 | MS_SF_ | MS_SF_/ MS_T x SF_ |
| T x Seed family | 307 | MS_T x SF_ | MS_T x SF_/MS_R_ |
| Residuals | 768 | MS_R_ |  |

Table S4: Summary of the analysis of the Selection Experiment using linear models. Shown are the degree of freedom, mean square values and calculation of F value for each explanatory variable. Degrees of freedom (DF) refer to n = 951 individuals, which differ according to the variable and the time of measurements.

| Source of variation | DF | Mean Square | F value |
| --- | --- | --- | --- |
| Block (B) | 3 | MS_B_ | MS_B_/MS_P_ |
| Species richness (SR) log-scale | 1 | MS_SR_ | MS_SR_/MS_P_ |
| Selection (SL) | 1 | MS_SL_ | MS_SL_/MS_SL X Sp-ID_ |
| SR x SL | 1 | MS_SR x SL_ | MS_SR x SL_/MS_SL X P_ |
| Functional group identity (FG-ID) | 3 | MS_FG-ID_ | MS_FG-ID_/MS_P_ |
| SR x FG-ID | 3 | MS_SR x FG-ID_ | MS_SR x FG-ID_/MS_Sp-ID x P_ |
| SL x FG-ID | 3 | MS_SL x FG-ID_ | MS_SL x FG-ID_/ MS_Sp-ID x P_ |
| Species identity (Sp-ID) | 5 | MS_Sp-ID_ | MS_Sp-ID_/ MS_Sp-ID x P_ |
| SR x Sp-ID | 5 | MS_SR x Sp-ID_ | MS_SR x Sp-ID_/ MS_Sp-ID x P_ |
| SL x Sp-ID | 5 | MS_SL x Sp-ID_ | MS_SL x Sp-ID_/MS_SF_ |
| Plot (P) | 47 | MS_P_ | MS_P_/MS_Sp-ID x P_ |
| Sp-ID x Plot | 7 | MS_Sp-ID x P_ | MS_Sp-ID x P_/MS_SF_ |
| SL x Plot | 48 | MS_SL X P_ | MS_SP_/MS_SF_ |
| Seed family | 204 | MS_SF_ | MS_SF_/ MS_R_ |
| Residuals | 614 | MS_R_ |  |

Table S5: Results of linear models of the *Community History Experiment* testing effects of canopy height of the surrounding vegetation as a covariate and the experimental factors on plant performance and trait expression. If variables were measured at different time points, it is indicated with t1= summer 2020, t2= spring 2021, and t3= summer 2021. Shown are F and P values. Abbreviations of variable names: LeafG= leaf greenness, C_Leaf_ = leaf carbon concentration, N_Leaf_ = leaf nitrogen concentration, SLA = specific leaf area.

| Source of variation | Plant biomass | | RGR_t3-t1 | | | | | | Plant height_t1 | | | | | | Plant height_t2 | | | | | | Plant height_t3 | | |  |  |  |  |  |
| --- | --- | --- | --- | --- | --- | --- | --- | --- | --- | --- | --- | --- | --- | --- | --- | --- | --- | --- | --- | --- | --- | --- | --- | --- | --- | --- | --- | --- |
|  | F | P | | F | | P | | | | F | | P | | | | F | | P | | | | F | P | |  |  |  |  |
| Block | 5.33 | 0.070 | | 189.75 | | 0.001 | | | | 19.23 | | 0.002 | | | | 31.79 | | <0.001 | | | | 26.36 | <0.001 | |  |  |  |  |
| Canopy height | 0.27 | 0.603 | | 1.83 | | 0.185 | | | | 59.38 | | <0.001 | | | | 284.94 | | <0.001 | | | | 47.86 | <0.001 | |  |  |  |  |
| Species richness (SR) (log) | 35.18 | <0.001 | | 16.37 | | 0.001 | | | | 13.25 | | 0.001 | | | | 19.88 | | <0.001 | | | | 9.33 | 0.006 | |  |  |  |  |
| Treatment | 3.42 | 0.040 | | 0.01 | | 0.991 | | | | 0.32 | | 0.726 | | | | 4.38 | | 0.015 | | | | 1.63 | 0.207 | |  |  |  |  |
| SR x Treatment | 1.09 | 0.342 | | 1.74 | | 0.190 | | | | 1.13 | | 0.328 | | | | 0.96 | | 0.387 | | | | 1.77 | 0.180 | |  |  |  |  |
| FG-ID | 27.79 | <0.001 | | 6.53 | | 0.009 | | | | 17.84 | | <0.001 | | | | 53.87 | | <0.001 | | | | 56.70 | <0.001 | |  |  |  |  |
| SR x FG-ID | 1.50 | 0.500 | | 891.00 | | 0.021 | | | | 13.20 | | 0.005 | | | | 3.05 | | 0.092 | | | | 4.33 | 0.322 | |  |  |  |  |
| Treatment x FG-ID | 3.50 | 0.379 | | 4.00 | | 0.349 | | | | 1.18 | | 0.425 | | | | 1.07 | | 0.453 | | | | 0.92 | 0.645 | |  |  |  |  |
| Species-ID | 34.33 | 0.125 | | 2280.00 | | 0.015 | | | | 25.65 | | 0.001 | | | | 40.27 | | <0.001 | | | | 5.44 | 0.303 | |  |  |  |  |
| SR x Species-ID | 0.50 | 0.707 | | 66.00 | | 0.087 | | | | 7.20 | | 0.018 | | | | 3.93 | | 0.043 | | | | 0.50 | 0.707 | |  |  |  |  |
| Treatment x Species-ID | 1.59 | 0.180 | | 0.40 | | 0.810 | | | | 0.86 | | 0.549 | | | | 1.61 | | 0.113 | | | | 1.65 | 0.162 | |  |  |  |  |
| Plot | 2.20 | 0.009 | | 1.74 | | 0.087 | | | | 2.50 | | <0.001 | | | | 2.90 | | <0.001 | | | | 3.13 | <0.001 | |  |  |  |  |
| Species-ID x Plot | 0.27 | 0.603 | | 0.01 | | 0.925 | | | | 1.07 | | 0.384 | | | | 1.20 | | 0.309 | | | | 0.89 | 0.351 | |  |  |  |  |
| Subplot | 2.92 | 0.055 | | 22.20 | | <0.001 | | | | 1.53 | | 0.137 | | | | 1.16 | | 0.337 | | | | 1.43 | 0.312 | |  |  |  |  |
| Species-ID x Subplot | 1.25 | 0.273 | | 0.12 | | 0.994 | | | | 1.98 | | 0.008 | | | | 1.90 | | 0.005 | | | | 1.78 | 0.084 | |  |  |  |  |
| SF | 1.61 | 0.003 | | 1.10 | | 0.324 | | | | 1.13 | | 0.166 | | | | 1.12 | | 0.173 | | | | 1.17 | 0.173 | |  |  |  |  |
| Treatment x SF | 0.76 | 0.984 | | 0.98 | | 0.551 | | | | 1.02 | | 0.403 | | | | 1.43 | | <0.001 | | | | 0.90 | 0.791 | |  |  |  |  |
| Source of variation | LeafG_t1 | | LeafG_t3 | | | | SLA | | | | | | N_Leaf_ | | | | | | C_Leaf_ | | | | |  | |  | |  |
|  | F | P | | F | P | | | F | | | P | | | F | | | P | | | F | | | P | |  | |  | |
| Block | 9.80 | 0.010 | | 42.61 | 0.002 | | | 34.1 | | | <0.001 | | | 30.57 | | | <0.001 | | | 7.73 | | | <0.001 | |  | |  | |
| Canopy height | 66.37 | <0.001 | | 23.84 | <0.001 | | | 303.5 | | | <0.001 | | | <0.01 | | | >0.999 | | | 8.85 | | | 0.004 | |  | |  | |
| Species richness (SR) (log) | 38.50 | <0.001 | | 44.15 | <0.001 | | | 9.7 | | | 0.003 | | | 17.59 | | | <0.001 | | | 2.42 | | | 0.126 | |  | |  | |
| Treatment | 0.84 | 0.434 | | 1.45 | 0.243 | | | 4.5 | | | 0.013 | | | <0.01 | | | >0.999 | | | 2.08 | | | 0.130 | |  | |  | |
| SR x Treatment | 0.16 | 0.849 | | 2.08 | 0.136 | | | 1.2 | | | 0.313 | | | 2.40 | | | 0.095 | | | 0.26 | | | 0.771 | |  | |  | |
| FG-ID | 101.88 | <0.001 | | 90.99 | <0.001 | | | 70.8 | | | <0.001 | | | 206.89 | | | <0.001 | | | 67.69 | | | <0.001 | |  | |  | |
| SR x FG-ID | 8.81 | 0.013 | | 0.53 | 0.698 | | | 1.0 | | | 0.439 | | | 2.22 | | | 0.163 | | | 2.05 | | | 0.185 | |  | |  | |
| Treatment x FG-ID | 1.12 | 0.446 | | 2.50 | 0.439 | | | 0.3 | | | 0.931 | | | 0.11 | | | 0.992 | | | 0.72 | | | 0.647 | |  | |  | |
| Species-ID | 6.60 | 0.022 | | 26.38 | 0.142 | | | 15.6 | | | 0.001 | | | 28.27 | | | <0.001 | | | 13.54 | | | 0.001 | |  | |  | |
| SR x Species-ID | 4.71 | 0.046 | | 2.65 | 0.398 | | | 1.1 | | | 0.440 | | | 1.33 | | | 0.341 | | | 1.11 | | | 0.426 | |  | |  | |
| Treatment x Species-ID | 3.16 | 0.003 | | 0.70 | 0.627 | | | 1.8 | | | 0.066 | | | 0.72 | | | 0.703 | | | 1.04 | | | 0.415 | |  | |  | |
| Plot | 3.09 | <0.001 | | 2.43 | 0.004 | | | 2.1 | | | 0.001 | | | 3.83 | | | <0.001 | | | 2.58 | | | <0.001 | |  | |  | |
| Species-ID x Plot | 1.23 | 0.300 | | 0.21 | 0.652 | | | 3.4 | | | 0.002 | | | 3.61 | | | 0.001 | | | 1.69 | | | 0.109 | |  | |  | |
| Subplot | 3.04 | 0.004 | | 1.59 | 0.249 | | | 1.6 | | | 0.072 | | | 1.02 | | | 0.497 | | | 0.58 | | | 0.971 | |  | |  | |
| Species-ID x Subplot | 0.59 | 0.913 | | 1.15 | 0.332 | | | 1.0 | | | 0.427 | | | 1.72 | | | 0.014 | | | 1.76 | | | 0.011 | |  | |  | |
| SF | 0.93 | 0.726 | | 1.11 | 0.263 | | | 1.1 | | | 0.329 | | | - | | | - | | | - | | | - | |  | |  | |
| Treatment x SF | 1.08 | 0.187 | | 1.23 | 0.049 | | | 0.9 | | | 0.843 | | | - | | | - | | | - | | | - | |  | |  | |

Table S6: Results of linear models for the *Selection Experiment* testing effects of canopy height of the surrounding vegetation height as a covariate and the experimental factors on trait performance and trait expression. If variables were measured at different time points, it is indicated with t1= summer 2020, t2= spring 2021, and t3= summer 2021. Shown are F and P values. Abbreviations of variable names: LeafG= leaf greenness, C_Leaf_ = leaf carbon concentration, N_Leaf_ = leaf nitrogen concentration, SLA = specific leaf area.

| Source of variation | Plant biomass | | RGR_t3-t1 | | Plant height_t1 | | Plant height_t2 | | Plant height_t3 | |
| --- | --- | --- | --- | --- | --- | --- | --- | --- | --- | --- |
|  | F | P | F | P | F | P | F | P | F | P |
| Block | 1.54 | 0.231 | 0.65 | 0.596 | 7.22 | <0.001 | 7.87 | <0.001 | 33.72 | <0.001 |
| Canopy height | 0.52 | 0.477 | 1.23 | 0.282 | 104.16 | <0.001 | 207.14 | <0.001 | 28.81 | <0.001 |
| Species richness (SR) (log) | 12.3 | 0.002 | 2.69 | 0.123 | 6.79 | 0.012 | 13.34 | 0.001 | 3.58 | 0.072 |
| Selection | 2.67 | 0.201 | 0.86 | 0.452 | 0.57 | 0.492 | 0 | 1 | 6.94 | 0.078 |
| SR x Selection | 0.52 | 0.477 | 3.7 | 0.071 | 1.4 | 0.242 | 0.31 | 0.58 | 1.43 | 0.243 |
| FG-ID | 16.26 | <0.001 | 1.63 | 0.232 | 6.77 | 0.001 | 33.27 | <0.001 | 46.16 | <0.001 |
| SR x FG-ID | 13.5 | 0.189 | 49 | 0.09 | 6.78 | 0.024 | 1.14 | 0.399 | 9.5 | 0.224 |
| Selection x FG-ID | 0.5 | 0.707 | 7.25 | 0.254 | 1.15 | 0.402 | 0.57 | 0.654 | 26.5 | 0.136 |
| Species-ID | 17.33 | 0.174 | 40.5 | 0.11 | 10.32 | 0.007 | 24.63 | <0.001 | 133 | 0.064 |
| SR x Species-ID | 0.5 | 0.707 | 2.25 | 0.426 | 3.97 | 0.066 | 3.14 | 0.084 | 1.5 | 0.5 |
| Species-ID x Selection | 1.99 | 0.12 | 1.44 | 0.244 | 2 | 0.095 | 1.29 | 0.271 | 1.33 | 0.267 |
| Plot | 6.91 | 0.293 | 11.54 | 0.227 | 1.96 | 0.202 | 1.79 | 0.215 | 32 | 0.139 |
| Species-ID x Plot | 0.66 | 0.417 | 0.41 | 0.523 | 3.75 | 0.001 | 3.09 | 0.004 | 0.17 | 0.684 |
| Plot x Selection | 1.27 | 0.198 | 0.83 | 0.65 | 1.36 | 0.069 | 1.88 | 0.001 | 1.75 | 0.026 |
| SF | 1.22 | 0.095 | 1.18 | 0.18 | 1.12 | 0.13 | 1.38 | 0.001 | 0.8 | 0.915 |
| Source of variation | LeafG_t1 | | Leaf G_t3 | | SLA | | N_Leaf_ | | C_Leaf_ | |
|  | F | P | F | P | F | P | F | P | F | P |
| Block | 1.14 | 0.345 | 5.38 | 0.006 | 11.68 | <0.001 | 16.36 | <0.001 | 4 | 0.013 |
| Canopy height | 78.72 | <0.001 | 2.3 | 0.142 | 268.55 | <0.001 | 3.27 | 0.077 | 1.59 | 0.213 |
| Species richness (SR) (log) | 35.92 | <0.001 | 33.87 | <0.001 | 5.2 | 0.027 | 31.79 | <0.001 | 10.8 | 0.002 |
| Selection | 0.49 | 0.522 | 0.88 | 0.418 | 0.18 | 0.687 | 0.83 | 0.403 | 2.92 | 0.148 |
| SR x Selection | 0.43 | 0.517 | 0.51 | 0.484 | 2.34 | 0.133 | 4.9 | 0.032 | 3.43 | 0.07 |
| FG-ID | 58.72 | <0.001 | 69.74 | <0.001 | 44.09 | <0.001 | 138 | <0.001 | 38.07 | <0.001 |
| SR x FG-ID | 6.83 | 0.023 | 20.33 | 0.155 | 1.02 | 0.439 | 7.78 | 0.012 | 0.37 | 0.778 |
| Selection x FG-ID | 1.55 | 0.295 | 12 | 0.2 | 0.72 | 0.569 | 1.56 | 0.283 | 3.19 | 0.093 |
| Species-ID | 7.89 | 0.014 | 36.93 | 0.12 | 11.03 | 0.003 | 69.07 | <0.001 | 14.59 | 0.001 |
| SR x Species-ID | 3.26 | 0.095 | 17.22 | 0.168 | 0.92 | 0.519 | 3.27 | 0.077 | 9.28 | 0.005 |
| Species-ID x Selection | 3.96 | 0.004 | 0.97 | 0.411 | 1.69 | 0.138 | 3.14 | 0.009 | 0.42 | 0.835 |
| Plot | 2.01 | 0.194 | 13.74 | 0.21 | 0.9 | 0.629 | 3.38 | 0.048 | 3.68 | 0.038 |
| Species-ID x Plot | 2.38 | 0.03 | 0.12 | 0.724 | 4.43 | <0.001 | 1.12 | 0.348 | 0.47 | 0.853 |
| Plot x Selection | 1.13 | 0.276 | 0.91 | 0.596 | 1.15 | 0.243 | 1.6 | 0.009 | 1.43 | 0.037 |
| SF | 1.11 | 0.148 | 1.85 | <0.001 | 0.94 | 0.717 | - | - | - | - |

Table S7: Results of Tukey’s HSD test used to identify differences among four functional groups in the *Community History Experiment*. Shown are mean values and standard error (SE) of measured values for each functional group; letters indicate statistically significant differences.

| Trait | Grasses | | | Legumes | | | | Small herbs | | | | Tall herbs | | | |
| --- | --- | --- | --- | --- | --- | --- | --- | --- | --- | --- | --- | --- | --- | --- | --- |
|  | Mean | SE | Group | | Mean | SE | Group | | Mean | SE | Group | | Mean | SE | Group |
| Survival rate_t1 | 0.949 | 0.009 | b | | 0.905 | 0.012 | ab | | 0.926 | 0.010 | b | | 0.861 | 0.018 | a |
| Survival rate_t2 | 0.728 | 0.017 | a | | 0.605 | 0.020 | a | | 0.756 | 0.016 | a | | 0.689 | 0.019 | a |
| Survival rate_t3 | 0.616 | 0.019 | ab | | 0.515 | 0.020 | ab | | 0.691 | 0.017 | b | | 0.491 | 0.021 | a |
| Flowering proportion | 0.373 | 0.022 | b | | 0.457 | 0.025 | b | | 0.575 | 0.020 | b | | 0.262 | 0.022 | a |
| Plant biomass (g) | 0.418 | 0.039 | a | | 2.617 | 0.264 | b | | 1.516 | 0.088 | b | | 0.563 | 0.064 | a |
| Relative growth rate t3_t1 | -0.002 | 0.000 | a | | 0.000 | 0.000 | b | | 0.000 | 0.000 | b | | 0.000 | 0.000 | b |
| Plant height_t1 (cm) | 15.657 | 0.300 | bc | | 20.729 | 0.464 | c | | 10.001 | 0.180 | a | | 13.516 | 0.407 | ab |
| Plant height_t2 (cm) | 35.386 | 0.620 | b | | 16.655 | 0.447 | a | | 15.318 | 0.330 | a | | 15.874 | 0.474 | a |
| Plant height_t3 (cm) | 24.535 | 0.600 | b | | 32.080 | 0.936 | b | | 11.994 | 0.289 | a | | 16.245 | 0.547 | a |
| Leaf greennes_t1 | 28.424 | 0.361 | a | | 49.578 | 0.400 | d | | 36.229 | 0.346 | c | | 32.512 | 0.543 | b |
| Leaf greenness_t3 | 24.154 | 0.398 | a | | 47.035 | 0.477 | d | | 33.032 | 0.347 | c | | 28.016 | 0.409 | b |
| SLA (mm^2^_leaf_ g^-1^_leaf_) | 23.527 | 0.270 | b | | 26.304 | 0.469 | c | | 17.465 | 0.297 | a | | 23.446 | 0.311 | b |
| N_Leaf_ | 18.224 | 0.389 | ab | | 43.723 | 0.581 | c | | 16.611 | 0.258 | a | | 20.991 | 0.453 | b |
| C_Leaf_ | 44.784 | 0.122 | b | | 48.137 | 0.281 | c | | 43.147 | 0.076 | a | | 45.450 | 0.144 | b |

Table S8: Results of Tukey’s HSD test used to identify differences among four functional groups in the *Selection Experiment*. Shown are mean values and standard error (SE) of measured values for each functional group; letters indicate statistically significant differences.

| Trait | Grasses | | | Legumes | | | | Small herbs | | | | Tall herbs | | | |
| --- | --- | --- | --- | --- | --- | --- | --- | --- | --- | --- | --- | --- | --- | --- | --- |
|  | Mean | SE | Group | | Mean | SE | Group | | Mean | SE | Group | | Mean | SE | Group |
| Survival rate_t1 | 0.900 | 0.015 | a | | 0.892 | 0.015 | a | | 0.934 | 0.011 | a | | 0.877 | 0.019 | a |
| Survival rate_t2 | 0.653 | 0.023 | a | | 0.589 | 0.024 | a | | 0.697 | 0.021 | a | | 0.662 | 0.022 | a |
| Survival rate_t3 | 0.514 | 0.024 | ab | | 0.464 | 0.024 | ab | | 0.677 | 0.021 | b | | 0.412 | 0.023 | a |
| Flowering proportion | 0.374 | 0.028 | ab | | 0.447 | 0.030 | ab | | 0.583 | 0.025 | b | | 0.247 | 0.024 | a |
| Plant biomass (g) | 0.316 | 0.036 | a | | 1.568 | 0.172 | b | | 1.299 | 0.091 | b | | 0.555 | 0.070 | a |
| Relative growth rate t3_t1 | -0.002 | 0.000 | a | | 0.000 | 0.000 | b | | 0.000 | 0.000 | b | | 0.000 | 0.000 | b |
| Plant height_t1 (cm) | 14.570 | 0.368 | bc | | 20.488 | 0.597 | c | | 9.876 | 0.206 | a | | 13.749 | 0.439 | ab |
| Plant height_t2 (cm) | 33.901 | 0.771 | b | | 16.741 | 0.592 | a | | 16.005 | 0.441 | a | | 15.469 | 0.497 | a |
| Plant height_t3 (cm) | 24.268 | 0.797 | b | | 32.692 | 1.078 | b | | 12.325 | 0.420 | a | | 17.402 | 0.631 | a |
| Leaf greennes_t1 (cm) | 27.079 | 0.461 | a | | 47.947 | 0.543 | c | | 35.817 | 0.418 | b | | 33.244 | 0.663 | b |
| Leaf greenness_t3 | 23.402 | 0.494 | a | | 45.787 | 0.620 | d | | 31.645 | 0.380 | c | | 27.144 | 0.437 | b |
| SLA (mm^2^_leaf_ g^-1^_leaf_) | 24.015 | 0.361 | bc | | 24.971 | 0.434 | c | | 16.957 | 0.293 | a | | 22.990 | 0.372 | b |
| N_Leaf_ | 18.110 | 0.501 | ab | | 42.489 | 0.849 | c | | 16.413 | 0.355 | a | | 20.785 | 0.509 | b |
| C_Leaf_ | 44.863 | 0.150 | b | | 47.453 | 0.386 | c | | 42.924 | 0.092 | a | | 45.556 | 0.209 | b |

Table S9: Results of Tukey’s HSD test used to identify differences in trait values among the three test environments of the ΔBEF Experiment. Shown are mean values and standard error (SE) of measured values for each functional group; letters indicate statistically significant differences.

| Trait | new plants, new soil | | | new plants, old soil | | | control | | |
| --- | --- | --- | --- | --- | --- | --- | --- | --- | --- |
|  | Mean | SE | Group | Mean | SE | Group | Mean | SE | Group |
| Survival_t2 | 0.687 | 0.016 | a | 0.750 | 0.015 | b | 0.660 | 0.016 | a |
| Survival_t3 | 0.589 | 0.017 | ab | 0.638 | 0.017 | b | 0.533 | 0.017 | a |
| Plant height_t2 (cm) | 22.948 | 0.560 | b | 19.366 | 0.504 | a | 20.673 | 0.547 | a |
| SLA (mm^2^_leaf_ g^-1^_leaf_) | 23.160 | 0.363 | b | 21.664 | 0.309 | a | 21.684 | 0.279 | a |
| N_Leaf_ | 24.697 | 0.767 | b | 24.362 | 0.753 | ab | 23.437 | 0.736 | a |
| C_Leaf_ | 45.453 | 0.193 | a | 45.038 | 0.179 | a | 45.201 | 0.163 | a |

| Trait | new plants, new sol | | | new plants, old soil | | | control | | |
| --- | --- | --- | --- | --- | --- | --- | --- | --- | --- |
|  | Mean | SE | Group | Mean | SE | Group | Mean | SE | Group |
| Canopy height_t1 | 19.024 | 0.389 | a | 17.809 | 0.389 | a | 17.992 | 0.398 | a |
| Canopy height_t2 | 37.985 | 0.673 | a | 32.396 | 0.627 | b | 33.182 | 0.600 | b |
| Canopy height_t3 | 26.130 | 0.441 | a | 24.098 | 0.488 | a | 25.737 | 0.567 | a |

Table S10: Results of Tukey’s HSD test used to identify differences in canopy height among treatments of the ΔBEF Experiment. Shown are mean values and standard error (SE) for canopy height measured at different time points, i.e. t1 = summer 2020, t2 = spring 2021, t3 = summer 2021. Letters indicate statistically significant differences.

Figure S1: Effects of sown species richness on (A) plant height and (B) leaf greenness measured in the growing season after planting the phytometers for the *Community History Experiment*. Solid colored lines represent a significant relationship at the functional-group level, while the solid black line represents the mean response across all species.

**
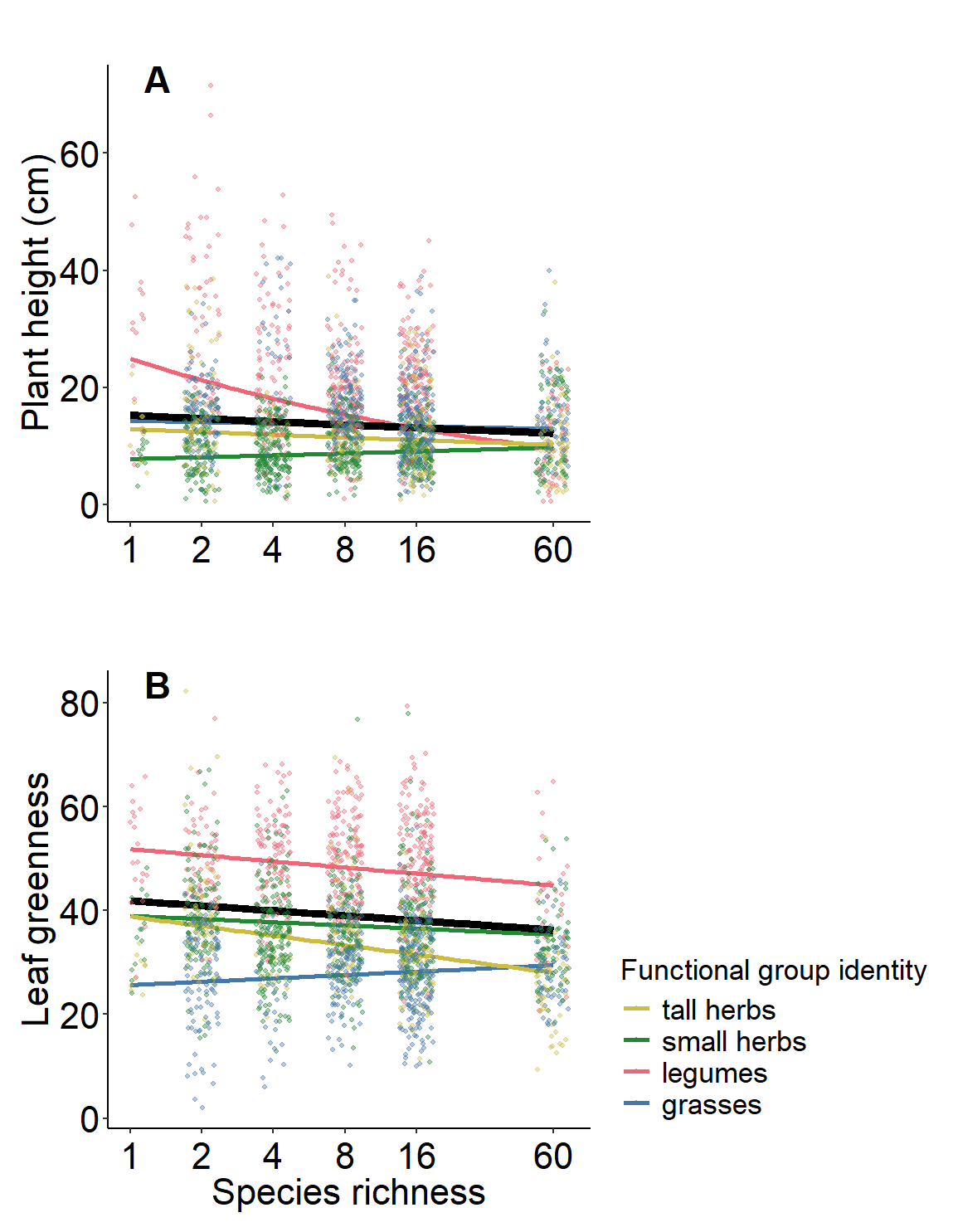
**

Figure S2: Effects of sown species richness on (A) plant individual biomass, (B) flowering proportion, (C) leaf greenness, (D) specific leaf area (SLA), (E) leaf nitrogen concentration (N_Leaf_), and (F) leaf carbon concentration (C_Leaf_) for the *Selection Experiment*. All variables were measured in the growing season after planting the phytometers. Solid colored lines represent a significant relationship at the functional-group level, while the solid black line represents the mean response across all species.


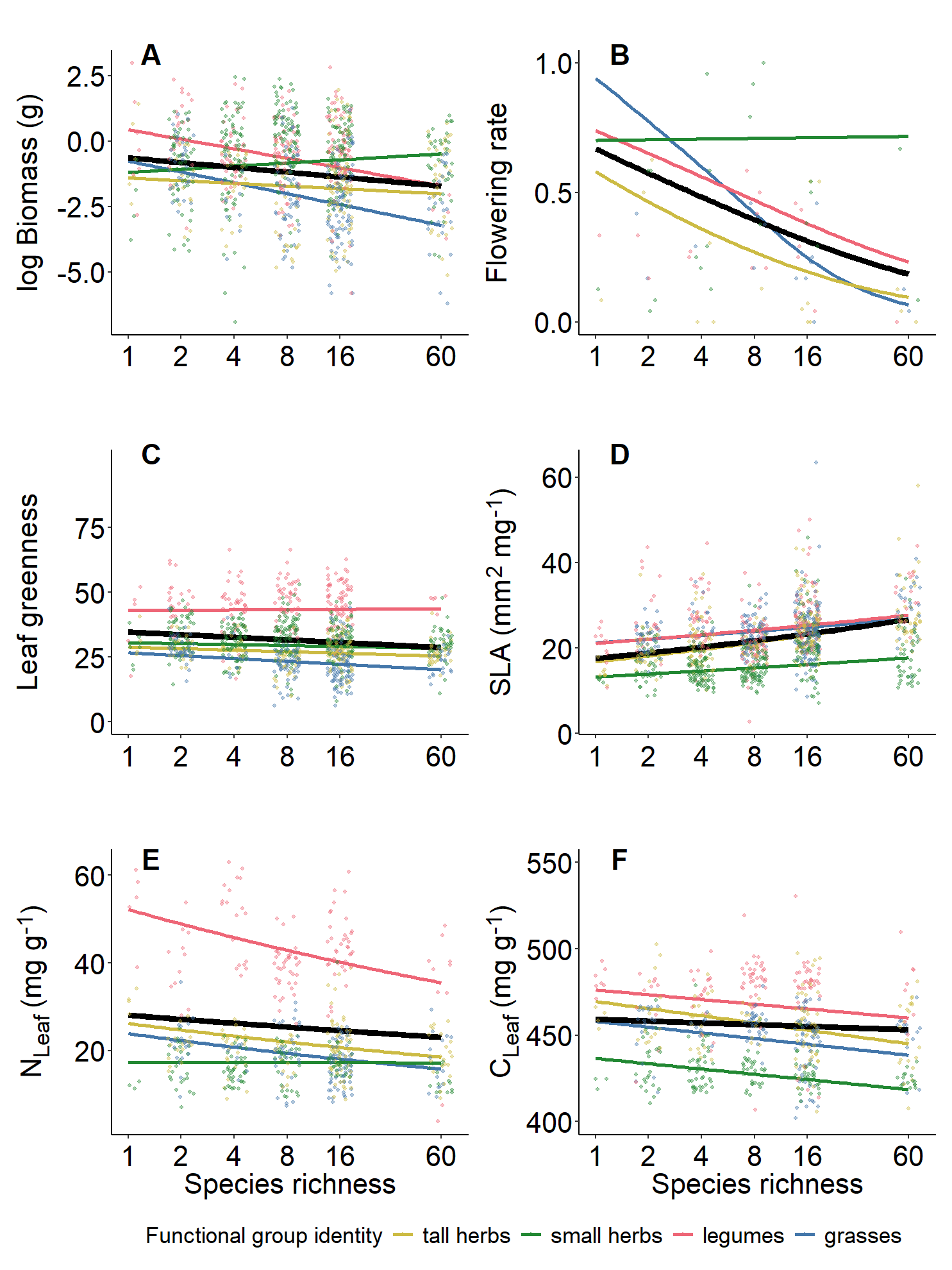


Figure S3: Effects of sown species richness on (A) leaf greenness (summer 2020) and (B) plant height (spring 2021) for the *Selection Experiment*. Solid colored lines represent a significant relationship at the functional-group level, while the solid black line represents the mean response across all species.

**
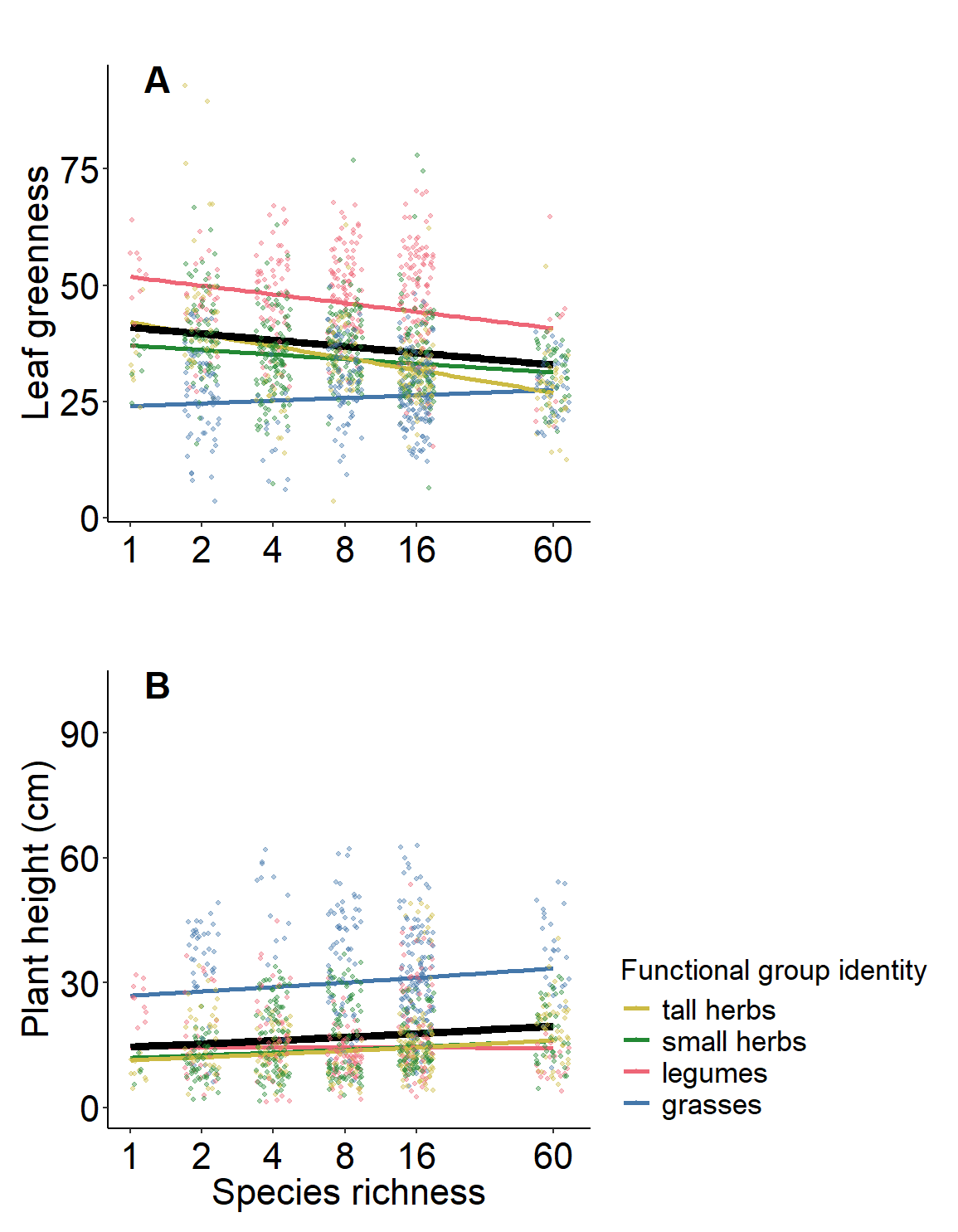
**

Figure S4: Effects of the treatment environments on (A) survival rate (spring 2021), (B) plant height (summer 2021) and (C) plant aboveground biomass in the growing season after planting the phytometers for the *Community History Experiment*. Solid colored lines represent a significant relationship at the treatment level. Shown are the different treatment environments of the ΔBEF Experiment. i.e. “new plants, new soil”, “new plants, old soil” and “old plants, old soil”.

**
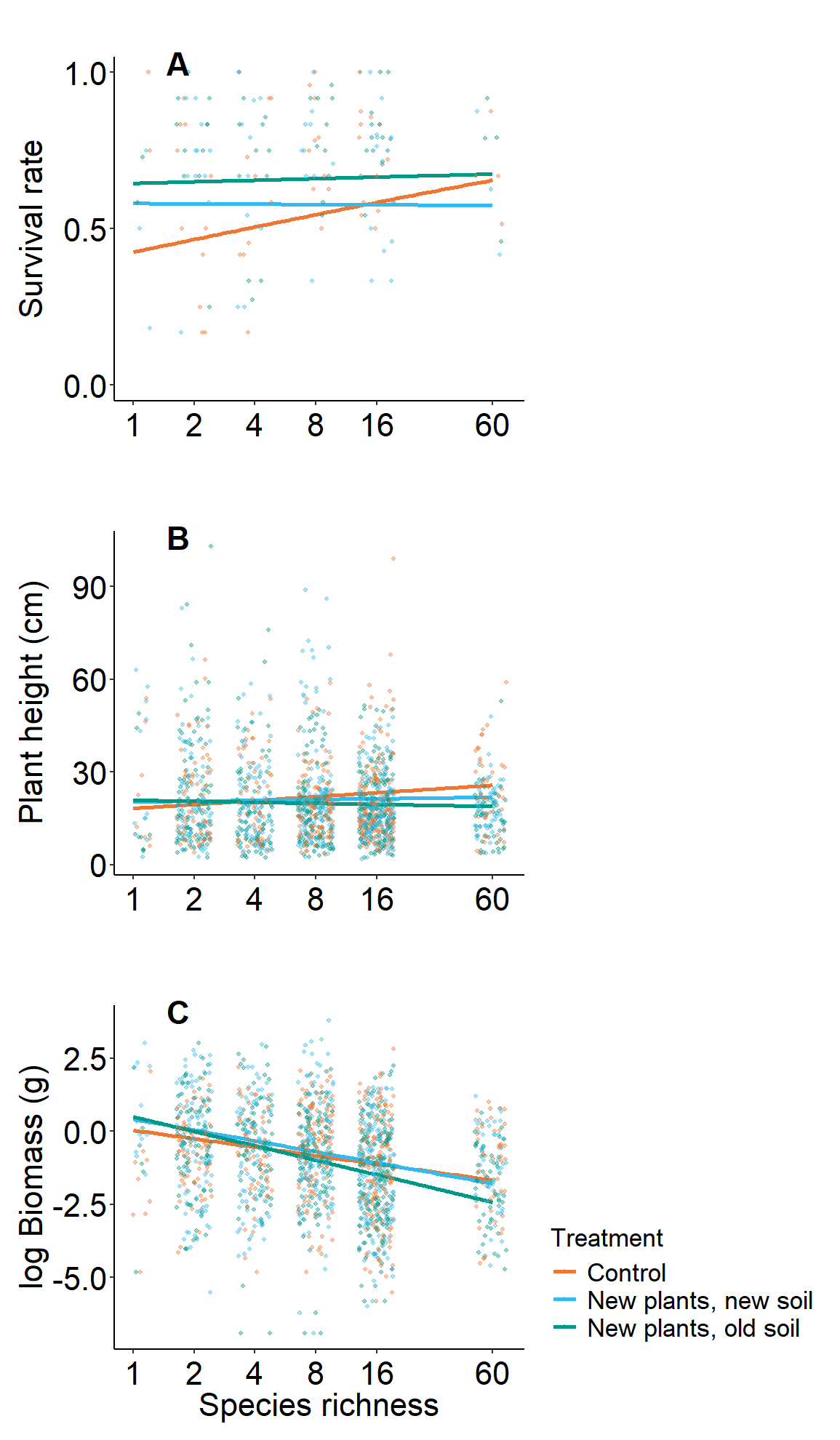
**

Figure S5: Plot means of canopy height for the three ΔBEF treatments measured (A) in summer 2020, (C) spring 2021, and (E) summer 2021, and subplot-means of vegetation height between the areas in which phytometers with and without selection history were transplanted (right panels) measured (B) in summer 2020, (D) spring 2021, and (F) summer 2021.


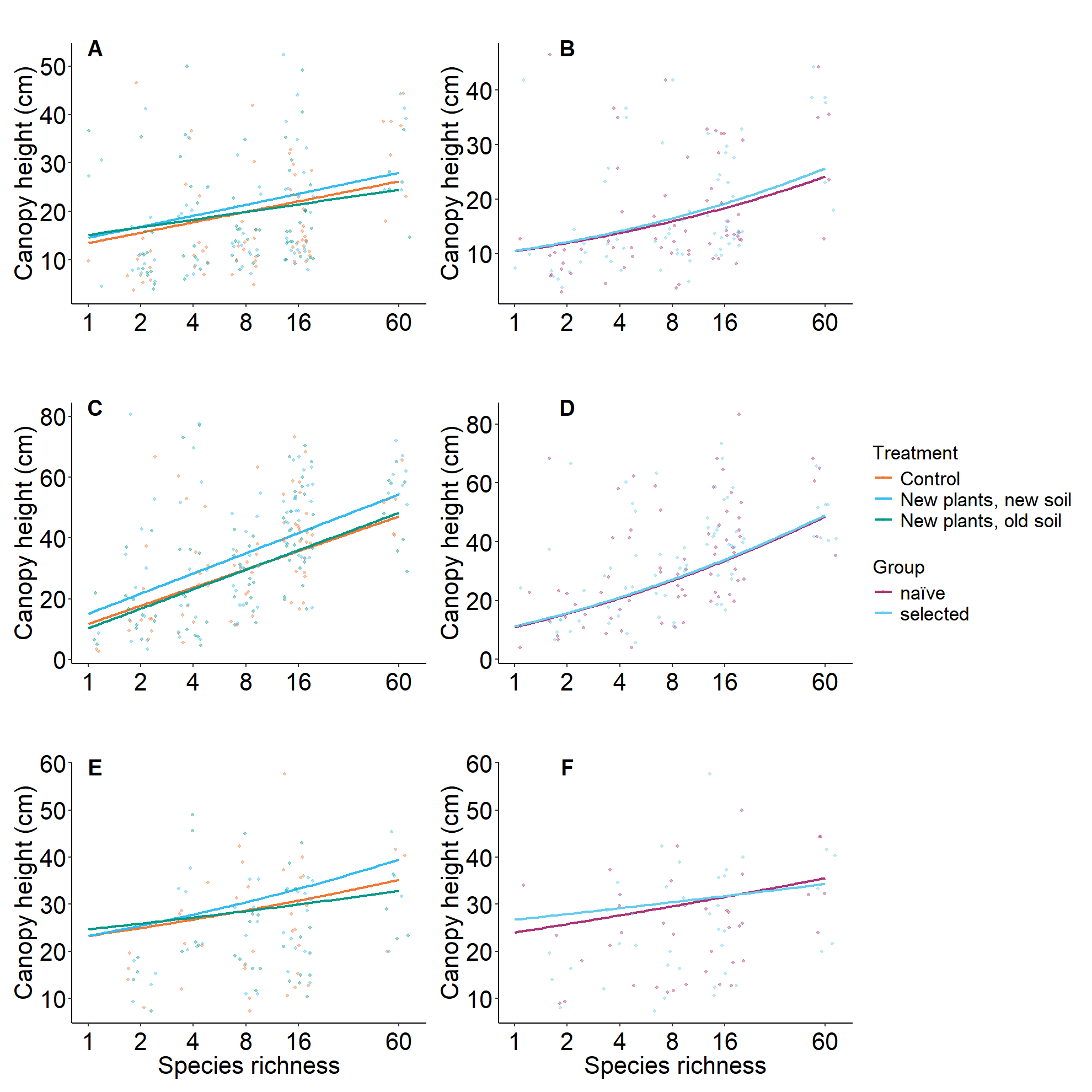

Supplement: Supplementary file 1 — Data S1. [file ECE3-15-e71117-s001.docx]
